# PM_2__.5_ Induce Endothelial‐Mesenchymal Transition and Cardiac Fibrosis via the NCOA4‐Mediated Ferritinophagy

**DOI:** 10.1002/advs.202507536

**Published:** 2025-09-15

**Authors:** Qinglin Sun, Mengyuan Wang, Lin Liu, Ruiyang Ding, Kanglin Yan, Shiqian Liu, Xiaoke Ren, Qing Xu, Zhiwei Sun, Qian Liu, Yi Yang, Junchao Duan

**Affiliations:** ^1^ Department of Toxicology and Sanitary Chemistry, School of Public Health Capital Medical University Beijing 100069 China; ^2^ Key Laboratory of Geographic Information Science of the Ministry of Education, School of Geographic Sciences East China Normal University Shanghai 200241 China; ^3^ State Key Laboratory of Environmental Chemistry and Ecotoxicology, Research Center for Eco‐Environmental Sciences Chinese Academy of Sciences Beijing 100085 China; ^4^ Laboratory for Clinical Medicine Capital Medical University Beijing 100069 China; ^5^ Beijing Key Laboratory of Environment and Aging Capital Medical University Beijing 100069 China; ^6^ Core Facilities for Electrophysiology, Core Facilities Center Capital Medical University Beijing 100069 China

**Keywords:** air pollution, cardiac fibrosis, endothelial‐to‐mesenchymal transition, ferritinophagy, particulate matter

## Abstract

Epidemiological evidence has indicated a strong association between fine particulate matter (PM_2.5_) exposure and adverse cardiac outcomes, including dysfunction and fibrosis. However, the underlying mechanisms remain unclear. In this study, the chemical species‐specific translocation of PM_2.5_ is investigated to the heart and its associated toxicological mechanisms. It is found that PM_2.5_‐derived iron (Fe)‐containing particles, particularly magnetite, are specifically enriched in the hearts of mice, with Fe content in individual particles increasing progressively along the path from the lungs through serum to the heart. Notably, molecular dynamics simulations demonstrated that Fe‐containing particles can form complexes with the key ferritinophagy regulator (nuclear receptor co‐activator 4 [NCOA4]), thereby altering its structure and function. Further analyses confirmed that PM_2.5_ upregulated NCOA4 expression in endothelial cells, which promoted the binding of transcription factor Kruppel‐like factor 5 to transforming growth factor beta 1 promoter, driving endothelial‐to‐mesenchymal transition (EndMT) in vitro and in vivo. Additionally, PM_2.5_‐treated endothelial cells facilitated the transformation of cardiac fibroblasts through paracrine signaling, leading to extracellular matrix production and cardiac fibrosis. Collectively, these findings reveal a previously unrecognized mechanism by which PM_2.5_‐derived Fe‐containing particles can trigger EndMT and cardiac fibrosis via ferritinophagy, with important implications for understanding the cardiovascular risks associated with air pollution.

## Graphical abstract

This study primarily reveals that PM_2.5_‐derived Fe‐containing particles, particularly magnetite, are specifically enriched in the heart. Here, they interact with NCOA4 in endothelial cells, disrupt iron homeostasis by enhancing ferritinophagy, and subsequently trigger the process of EndMT through the NCOA4/KLF5 pathway. These molecular events promote cardiac fibroblast activation, leading to cardiac fibrosis. Thus, targeting NCOA4‐mediated ferritinophagy signaling may mitigate PM_2.5_‐associated cardiovascular injury.

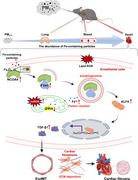

## 1 Introduction

According to the latest data released by the American Heart Association, cardiovascular diseases (CVDs) remain the leading cause of death worldwide, accounting for approximately 19.9 million deaths annually. Air pollution is recognized as a major environmental health risk for CVDs, contributing to 28% of premature deaths from ischemic heart disease and 27% from stroke.^[^ ^1^ ^]^ Among all attributable risk factors, fine particulate matter (PM_2.5_) were the largest contributor to the global burden of disease from air pollution in 2021, being responsible for 58% of premature deaths annually.^[^ ^1^, ^2^ ^]^ It is well documented that PM_2.5_ have been epidemiologically associated with adverse cardiovascular events, particularly increased hospital admissions for ischemic heart disease, heart failure, and arrhythmia, thereby representing a persistent threat to cardiac function.^[^ ^3^, ^4^ ^]^ Cardiac fibrosis is a critical pathological feature associated with various CVDs. This condition is characterized by excessive collagen deposition and stiffening of the heart tissue, which disrupts normal cardiac function and leads to heart failure.^[^ ^5^, ^6^ ^]^ Notably, although chronic exposure to PM_2.5_ has been shown to induce cardiac fibrosis and dysfunction,^[^ ^7^, ^8^ ^]^ the precise mechanisms underlying this association remain unclear.

Our previous study revealed that inhaled air pollution‐derived iron (Fe)‐rich particles, which pose a greater health risk than other particles, could enter the circulatory system, as demonstrated by chemical multi‐fingerprinting techniques.^[^ ^9^, ^10^ ^]^ More importantly, Fe‐rich particles were able to cross the endothelial cells and induce cardiac injury in human subjects after exposure to ambient particulate matter.^[^ ^11^ ^]^ Given the critical role of iron homeostatic imbalance in cardiac pathology, we hypothesized that Fe‐containing particles from PM_2.5_ could actively disrupt iron regulation and impair cardiac function. Ferritinophagy, a selective autophagic process that mediates intracellular iron release and is governed by nuclear receptor co‐activator 4 (NCOA4), ^[^ ^12^ ^]^ has emerged as a key regulator of iron balance and an initiating factor in cardiovascular injury.^[^ ^13^, ^14^, ^15^ ^]^ Endothelial‐to‐mesenchymal transition (EndMT) has also been identified as a major contributor to cardiac fibrosis, characterized by the loss of junctional proteins such as vascular endothelial cadherin (VE‐cadherin) and cluster of differentiation 31 (CD31) and the acquisition of mesenchymal traits including alpha‐smooth muscle actin (α‐SMA), vimentin, and fibronectin.^[^ ^16^, ^17^, ^18^ ^]^ Iron overload is increasingly recognized as an inducer of EndMT, promoting endothelial oxidative stress and triggering transcriptional reprogramming toward a mesenchymal phenotype. For instance, transforming growth factor beta 1 (TGF‐β1), a central mediator of EndMT in fibrotic hearts, can be potentiated by iron‐catalyzed reactive oxygen species.^[^ ^16^, ^19^, ^20^ ^]^ These findings suggest that iron‐driven EndMT substantially contributes to cardiac fibrotic remodeling and support the premise that iron overload–mediated induction of EndMT represents a critical step in fibrogenesis. Therefore, it is necessary to clarify how ferritinophagy is influenced by PM_2.5_‐derived Fe‐containing particles and whether it functions as a mechanistic bridge between PM_2.5_ exposure and cardiac fibrosis.

Based on the issues outlined above, a comprehensive study was conducted to investigate the effects and underlying mechanisms of PM_2.5_ exposure on cardiac fibrosis. This whole‐chain study was designed in a logical sequence, ranging from component identification, quantification, and characterization to molecular simulation and toxicological assessment. The specific objectives were as follows: (1) to examine the accumulation of PM_2.5_‐derived Fe‐containing particles in the heart using single‐particle inductively coupled plasma time‐of‐flight mass spectrometry (spICP‐TOF‐MS); (2) to explore the interactions of these particles with NCOA4 through molecular dynamics simulations; (3) to determine the role of NCOA4‐mediated ferritinophagy in promoting EndMT in endothelial cells and cardiac fibrosis; and (4) to evaluate the in vivo effects of PM_2.5_‐exposure on cardiac dysfunction and fibrosis in endothelial‐specific *Ncoa4* knockout mice. Our findings provide valuable mechanistic insights into the pathophysiology of PM_2.5_‐related cardiac injury and highlight potential therapeutic targets for mitigating the cardiovascular risks associated with air pollution exposure.

## 2 Results

### 2.1 Characterization of Fe‐Containing Particles in PM_2.5_ and In Vivo

**Figure 1:**
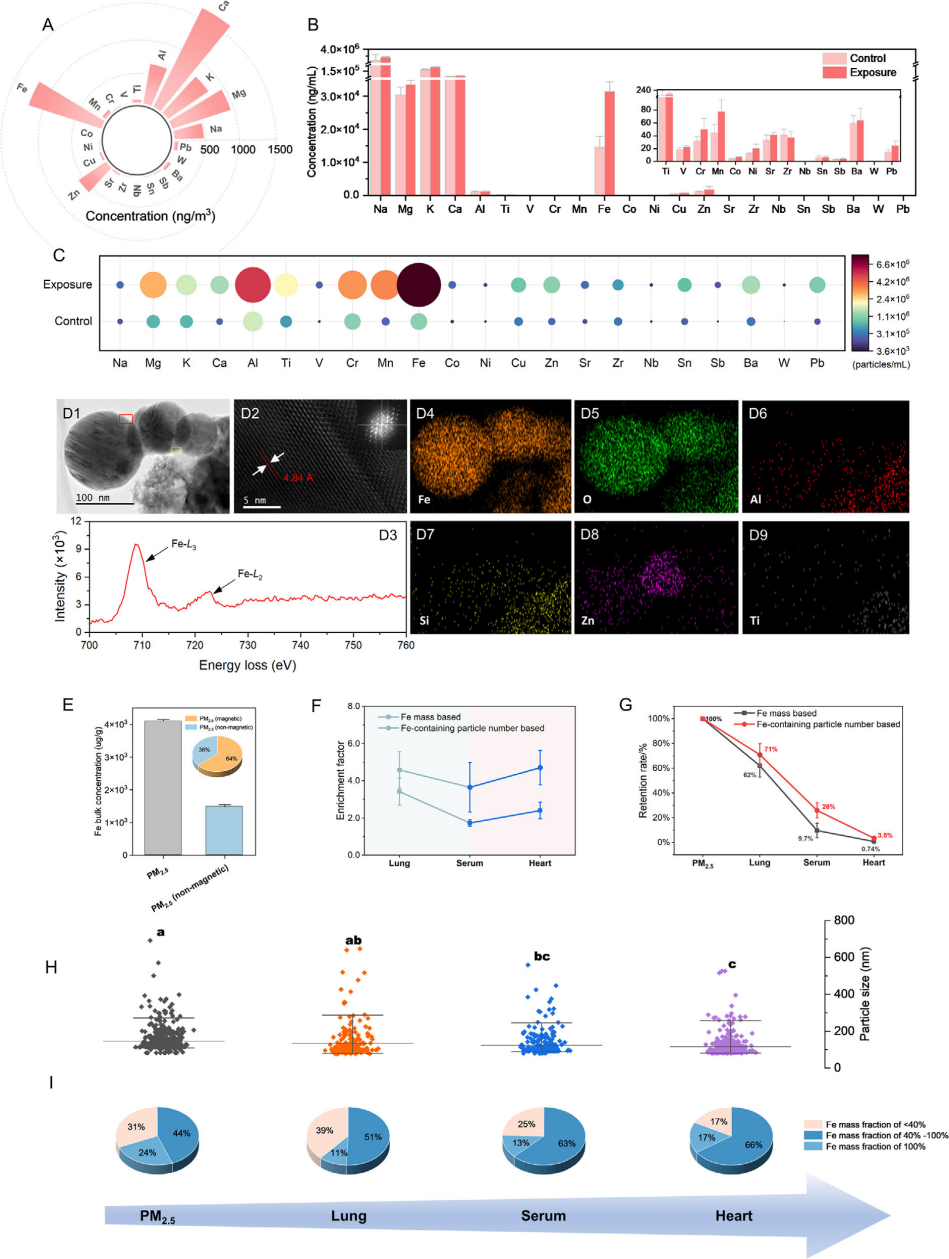
Characteristics of Fe‐containing particles in PM_2.5_ and in vivo. A) The distribution of 22 metal concentrations in PM_2.5_. B) The distribution of 22 metal concentrations in serum, with a comparison between the exposed and the control group. C) Number concentrations of the 22 metal‐containing particles in serum. D1) TEM image of magnetite particles in heart tissue. (D2) High‐resolution TEM image of the red square marked area and it's reduced Fast Fourier Transform (FFT) image. (D3) Electron energy loss spectroscopy (EELS) of the yellow square marked area in (D1). (D4–9) Energy dispersive X‐ray (EDX) mapping for the selected particles in (D1). E) Fe concentrations in total PM_2.5_ and non‐magnetic particles from PM_2.5_, with Fe concentration contributions demonstrated in the pie chart. F) Enrichment factors of Fe‐containing particles in lung, serum and heart of exposed mice compared to controls, based on Fe mass or the particle number. G) Fe‐containing particles retention rates based on Fe mass or the particle number. H) Particle size of Fe‐containing particles in PM_2.5_ and exposed mice lung, serum and heart tissues. I) Number proportions of Fe‐containing particles based on the Fe mass fractions in individual particles in PM_2.5_, lung, serum, and heart. n=8.

We first measured the concentrations of 22 metals in PM_2.5_ using inductively coupled plasma mass spectrometry (ICP‐MS). Among these metals, Fe had a concentration of 1287 ± 17 ng m^−3^, accounting for approximately 18% of the total concentration of the 22 metals (**Figure** **1**A). After 90 days of PM_2.5_ inhalation, the average Fe concentration in the serum of C57BL/6J mice was 3.1 × 10^4^ ± 6.0 × 10^3^ ng mL^−1^, which was lower than that of major elements of the Earth's crust, such as Na, K, Ca, and Mg, but significantly higher than that of other metals (Figure 1B). This distribution pattern was consistent with that reported in human serum.^[^ ^21^ ^]^ Notably, Fe exhibited the highest enrichment factor (defined as the ratio of elemental concentrations in exposed mice to those in controls) in serum (Figure S1, Supporting Information). Moreover, spICP‐TOF‐MS analysis revealed that Fe‐containing particles had the highest number concentration among all metal‐containing particles detected in the serum of exposed mice (Figure 1C), and these particles also displayed the highest enrichment factor. Collectively, these results suggested that Fe‐containing particles from PM_2.5_ were more likely to enter the bloodstream than other metal‐containing particles.

Various Fe‐containing particles, such as magnetite, hematite, and ilmenite, were observed in PM_2.5_, lung, serum, and heart tissues using transmission electron microscopy (TEM). Among these, magnetite particles were identified more frequently than other types (Figure S2, Supporting Information). Under identical imaging and sampling conditions, the substantially greater number of TEM fields in which magnetite was detected supports its higher detection rate. Figure 1D1 shows a typical magnetite aggregate with individual particle sizes ranging from 70 to 130 nm identified in the heart tissue of mice. The Fe/O ratio (0.75–0.78) in these Fe‐ and O‐rich spherical particles (Figure 1D4, 5; Figure S3A1, B1, B4, C1, C4, C5, Supporting Information) and their *d‐*spacings (Figure 1D2; Figure S3A3, C3, Supporting Information) aligned with those of magnetite crystals. Additionally, electron energy loss spectroscopy (EELS) (Figure 1D3; Figure S3B3, Supporting Information) revealed an Fe‐*L*_3_ edge absorption at 709.2 eV and broad peaks of Fe‐*L*_2_ edge absorption. The integrated areas of the *L*_3_/*L*_2_ ratio (≈5.1) further confirmed the presence of magnetite particles in the heart tissue of mice exposed to PM_2.5_. These magnetite particles were also found to be associated with other elements, including Al, Si, Mg, Mn, Ti, and Zn (Figure 1D4–9; Figure S3B4, C6, 7, Supporting Information).

### 2.2 Enrichment of Fe‐Containing Particles throughout the Exposure Route In Vivo

To trace the Fe‐containing particles in vivo, we used a cyclic magnetic extraction (CME) system to isolate these particles from various mouse tissues.^[^ ^22^ ^]^ A considerable proportion of Fe‐containing particles was magnetic (e.g., magnetite), accounting for approximately 64% of the total Fe mass in PM_2.5_ (Figure 1E), and these particles could be efficiently extracted using magnetic separation. As a representative type of Fe‐containing particles, magnetite particles in lung, serum, and heart tissues exhibited enrichment factors of 3.4, 1.7, and 2.4, respectively, based on their Fe concentrations in PM_2.5_‐exposed mice compared with controls (Figure 1F). Moreover, the contribution of Fe to the total concentrations of the 22 detected elements in magnetite particles was 51% for PM_2.5_, increasing from 43% in lung to 65% in serum, and reaching 71% in heart (Figure S3D1–4), suggesting an increasing abundance of Fe‐containing particles along the exposure route from lung to heart.

The spICP‐TOF‐MS technique enables the simultaneous measurement of multiple elements in individual particles, thereby providing the elemental composition of Fe‐containing particles on a particle‐by‐particle basis (Table S2, Supporting Information). According to spICP‐TOF‐MS analysis, magnetite particle number concentrations demonstrated a more pronounced enrichment of Fe‐containing particles compared with enrichment based on Fe concentrations, with enrichment factors of 4.5, 3.6, and 4.7 in lung, serum, and heart tissues of exposed mice, respectively (Figure 1F). The higher enrichment factors of magnetite particles by number compared with those by mass suggest greater enrichment of smaller Fe‐containing particles in vivo, particularly in the hearts of mice. Based on Fe mass in PM_2.5_ and in vivo distribution after 90 days of exposure, 62% of inhaled magnetite particles were retained in the lung, 9.7% in blood, and 0.74% in the heart (Figure 1G). When considering the number of these Fe‐containing particles, the retention rates increased to 71% in the lung, 26.4% in blood, and 3.5% in the heart (Figure 1G), indicating that a large number of minute Fe‐containing particles can enter the bloodstream and subsequently be transported into the heart.

Interestingly, the particle size of magnetite particles, as calculated from spICP‐TOF‐MS analysis, showed a significant decreasing trend from PM_2.5_ to lung, serum, and heart tissues in the PM_2.5_‐exposed group, with average sizes of 146 nm, 137 nm, 124 nm, and 115 nm in PM_2.5_, lung, serum, and heart, respectively (Figure 1H). All of these particle sizes were significantly larger than those observed in controls (Figure S3E, Supporting Information). In addition, these Fe‐containing particles were primarily Fe‐rich, with Fe mass fractions greater than 40% in individual particles from PM_2.5_ to in vivo. Among them, single‐metal Fe‐containing particles, in which only Fe was detected by spICP‐TOF‐MS (with an Fe mass fraction of 100%), accounted for approximately 11‐17% of all magnetite particles in mouse tissues by number. Moreover, the contribution of these Fe‐rich particles to the total number of magnetic particles increased in vivo, with 68% in PM_2.5_, 62% in lung, 76% in serum, and 83% in heart (Figure 1I), confirming the enrichment of Fe‐containing particles with higher abundance and smaller sizes in heart tissue.

### 2.3 Molecular Docking Simulation of Fe_3_O_4_ and NCOA4 Interaction

**Figure 2:**
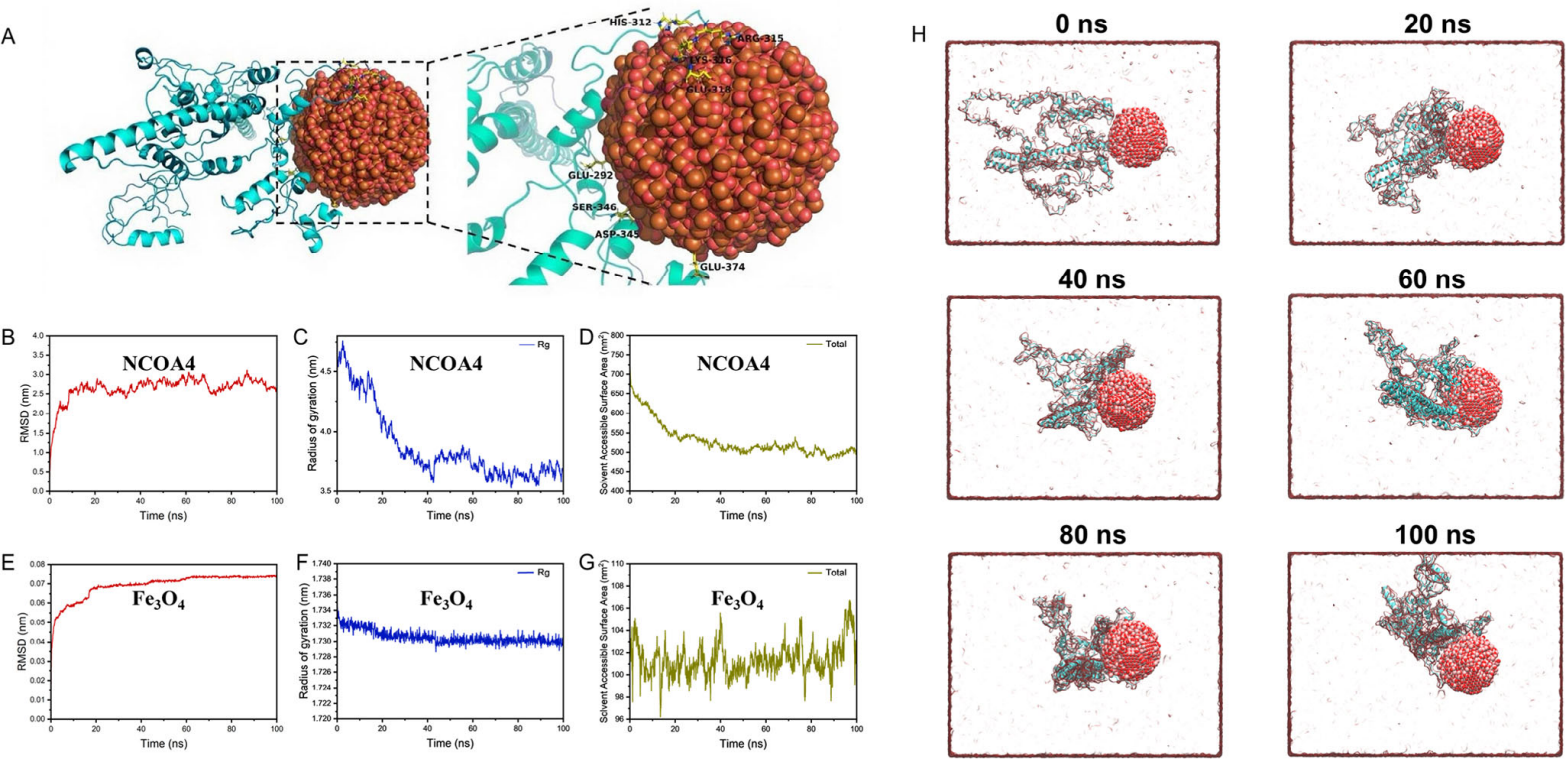
Molecular docking and molecular dynamics simulations to validate the interaction between Fe_3_O_4_ and NCOA4. A) Binding sites for the interaction of Fe_3_O_4_ with NCOA4. B–D) Root mean square deviation (RMSD), radius of gyration (Rg), and solvent‐accessible surface area (SASA) of NCOA4 in the course of an interaction. E–G) RMSD, Rg, and SASA of Fe_3_O_4_ in the course of an interaction. H) Visualization images of the interactions.

To investigate the potential molecular initiation event, we examined the interaction between Fe‐containing particles and iron metabolism regulatory proteins, using magnetite (Fe_3_O_4_) nanoparticles and NCOA4 as models in molecular dynamics simulations, since magnetite particles were the most frequently identified Fe‐containing particles in vivo. Conformational analysis demonstrated that Fe_3_O_4_ nanoparticles maintained a stable spherical structure in aqueous solution (Figure S4A, Supporting Information). Minimal fluctuations in root mean square deviation (RMSD), radius of gyration (Rg), and solvent‐accessible surface area (SASA) further confirmed their structural stability (Figure S4B, C, Supporting Information). In the simulation, NCOA4 was depicted as a green helix, while Fe_3_O_4_ nanoparticles appeared as red spheres, forming a stable interaction interface. Key interacting residues included His312, Arg315, Lys316, Glu318, and Glu374 (**Figure** **2**A). Molecular mechanics/poisson–boltzmann surface area (MM‐PBSA) analysis estimated the binding free energy of Fe_3_O_4_ with NCOA4 as ‐3100.795 ± 259.108 kJ mol^−1^, indicating a strong affinity.

Electrostatic interactions played a predominant role in binding, as Fe_3_O_4_ nanoparticles preferentially interacted with charged amino acids of NCOA4, whereas nonpolar amino acids contributed to the interaction through van der Waals forces. The electrostatic interaction energy (Coul‐SR) exhibited a rapid initial decrease and stabilized at approximately ‐2500 kJ mol^−1^, confirming its role as the primary driving force. In contrast, the van der Waals interaction energy (LJ‐SR) remained relatively stable and contributed to a lesser extent (Figure S4E, Supporting Information). Structural dynamics analysis revealed conformational adjustments in NCOA4 upon binding to Fe_3_O_4_ nanoparticles. The RMSD of NCOA4 increased initially before stabilizing, Rg gradually decreased, and SASA significantly declined, suggesting that NCOA4 adopted a more compact conformation with reduced solvent exposure (Figure 2B–D). Conversely, Fe_3_O_4_ nanoparticles displayed high structural stability, with negligible fluctuations in RMSD, Rg, and SASA (Figure 2E–G). In summary, Fe_3_O_4_ nanoparticles and NCOA4 formed a stable complex primarily driven by electrostatic interactions. The binding process induced structural rearrangements in NCOA4, while Fe_3_O_4_ nanoparticles maintained their integrity throughout the simulation (Figure 2H and Video S1, Supporting Information).

### 2.4 PM_2.5_ Induced Ferritinophagy and EndMT in HAECs

Since molecular docking simulations demonstrated that Fe‐containing particles in PM_2.5_ could strongly interact with NCOA4, we proposed a mechanistic hypothesis that PM_2.5_ binding to NCOA4 promotes cellular ferritinophagy and iron overload. As the first point of contact between circulating PM_2.5_ particles and the cardiovascular system, endothelial cells play a critical role in facilitating particle entry into cardiac tissue. Therefore, studying endothelial cells is essential for understanding the mechanisms underlying PM_2.5_‐induced cardiac effects. The enrichment factors of Fe‐containing particles were increased in human aortic endothelial cells (HAECs) compared with the control group (Figure S5A, Supporting Information), indicating that a substantial number of Fe‐containing particles entered the cells. A proliferative effect of HAECs was observed at a concentration of 12.5 µg mL^−1^, which was selected as the PM_2.5_ exposure concentration for subsequent experiments (Figure S5B, Supporting Information).

**Figure 3:**
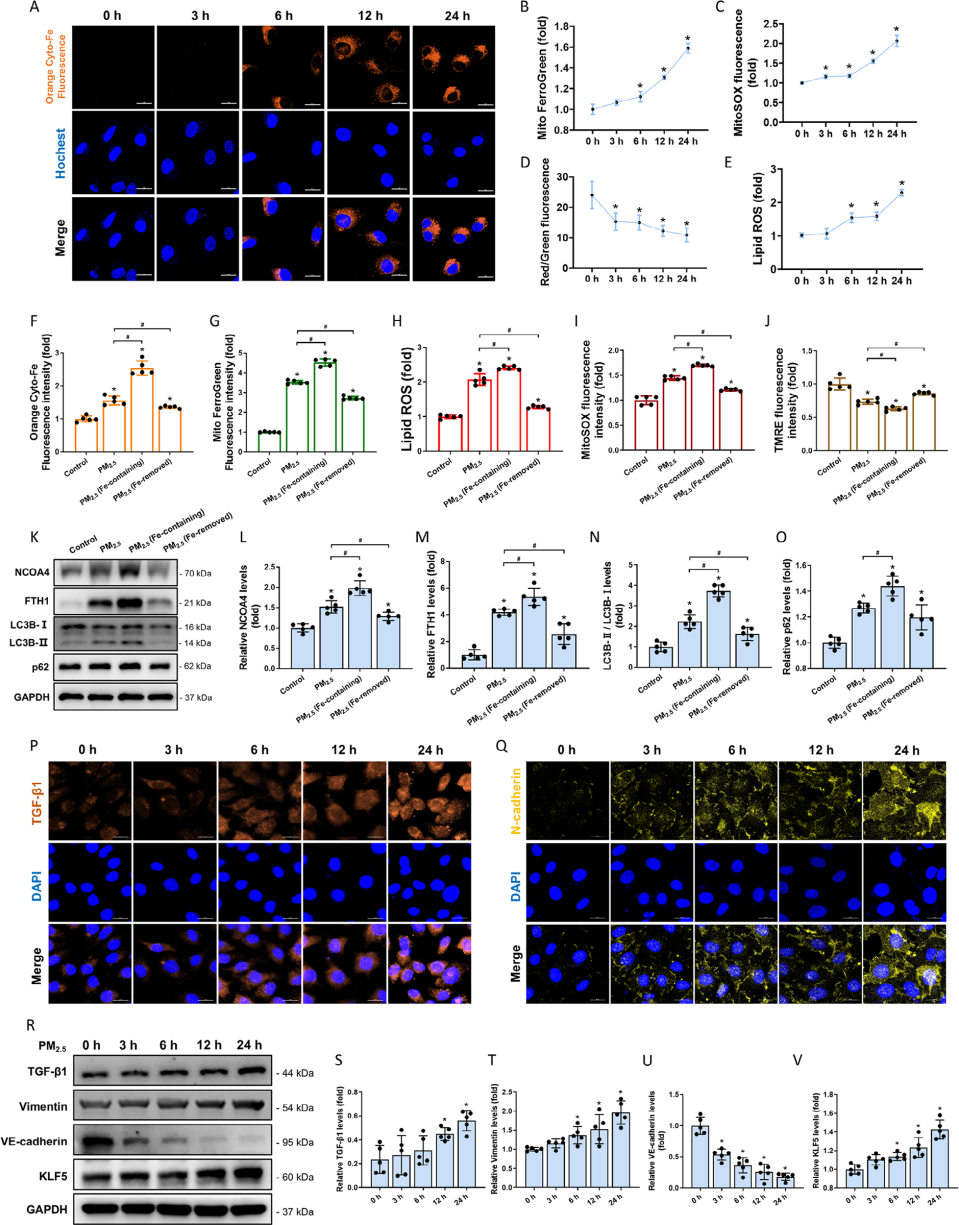
PM_2.5_ induced ferritinophagy and EndMT in HAECs. A) Representative fluorescence images of intracellular Fe^2+^ (scale bar: 20 µm). B–E) Analysis of mitochondrial Fe^2+^, mitochondrial ROS, mitochondrial membrane potential and lipid ROS by flow cytometry. F–J) Analysis of intracellular Fe^2+^, mitochondrial Fe^2+^, lipid ROS, mitochondrial ROS and mitochondrial membrane potential by flow cytometry. K) Representative Western blot pictures. PM_2.5_ (Fe‐removed): the fraction remaining after removal of the majority of Fe‐containing particles from PM_2.5_. L–O) Semi‐quantitative analysis of NCOA4, FTH1 and LC3B‐II/LC3B‐I and p62. P, Q) Representative immunofluorescence images of TGF‐β1 and N‐cadherin proteins (scale bar: 20 µm). R) Representative Western blot pictures. S–V) Semi‐quantitative analysis of TGF‐β1, N‐cadherin, VE‐cadherin and KLF5. n=5. All data were expressed as mean ± standard deviation. *p < 0.05, ^#^ *p* < 0.05.

Intracellular and mitochondrial Fe^2+^ levels were markedly increased in HAECs after exposure to PM_2.5_, suggesting that PM_2.5_ caused iron overload in a dose‐dependent manner (**Figure** **3**A, B; Figure S5C, D, Supporting Information). The elevated Fe^2+^ induced the Fenton reaction, which impaired mitochondrial structure and function, resulting in decreased mitochondrial membrane potential (MMP) and increased mitochondrial reactive oxygen species (mtROS) (Figure 3C, D; Figure S5E, F, Supporting Information). In addition, PM_2.5_ exposure increased lipid ROS levels in HAECs in a time‐dependent manner (Figure 3E; Figure S5G, Supporting Information). Moreover, PM_2.5_ upregulated the expression of NCOA4 and FTH1, as well as the autophagy markers LC3B‐II and p62 (Figure S5H–M, Supporting Information). After magnetic fractionation of PM_2.5_, both the magnetic fraction (i.e., magnetite particles) and the non‐magnetic fraction caused upregulation of Fe^2+^, lipid ROS, and mtROS levels, along with a reduction in MMP in HAECs (Figure 3F–J). However, magnetite particles, as typical Fe‐containing particles, exerted greater effects on ferritinophagy‐related changes than PM_2.5_ (Figure 3K–O). These findings suggested that Fe‐containing particles in PM_2.5_ played a critical role in PM_2.5_‐induced ferritinophagy.

Following treatment with PM_2.5_ for 3 h, 6 h, 12 h, and 24 h, immunofluorescence and Western blot assays demonstrated that PM_2.5_ significantly upregulated TGF‐β1, vimentin and N‐cadherin protein levels while reducing VE‐cadherin expression. These changes became more pronounced with increasing PM_2.5_ exposure duration (Figure 3P–U). These results indicate that PM_2.5_ exposure induced EndMT in HAECs in a time‐dependent manner. Moreover, PM_2.5_ significantly increased the expression of the transcription factor Kruppel‐like factor 5 (KLF5) (Figure 3V). In addition, PM_2.5_ increased TGF‐β1 content in the conditioned medium of HAECs in a time‐dependent manner (Figure S5N, Supporting Information). When the conditioned medium was applied to cardiac fibroblasts (HEH), PM_2.5_‐activated HAECs stimulated HEH, leading to significantly increased expression of fibrotic markers α‐SMA, Col I, and Col III (Figure S5O–S, Supporting Information).

### 2.5 Iron Chelation and NCOA4 Knockdown Mitigate PM_2.5_‐Triggered Ferritinophagy and EndMT

To validate the role of iron overload in PM_2.5_‐induced ferritinophagy and EndMT, HAECs were pretreated with 50 µM deferoxamine (DFO) for 2 h before PM_2.5_ exposure (Figure S6A, Supporting Information). DFO pretreatment reduced PM_2.5_‐induced accumulation of both total intracellular and mitochondrial Fe^2+^ levels (Figure S6B, C, Supporting Information). Correspondingly, Western blot analysis showed marked attenuation of NCOA4 and FTH1 upregulation in DFO‐treated cells (Figure S6D–F, Supporting Information). Functionally, DFO abolished PM_2.5_‐driven HAEC migration in scratch assays (Figure S6G, H, Supporting Information), and immunofluorescence revealed that DFO prevented the upregulation of TGF‐β1 and N‐cadherin (Figure S6, Supporting Information). Finally, conditioned medium from DFO‐pretreated HAECs failed to stimulate HEH proliferation, demonstrating that iron chelation disrupted the paracrine activation of fibroblasts (Figure S6M, N, Supporting Information). Together, these findings confirmed that iron overload played a critical role in PM_2.5_‐triggered ferritinophagy, EndMT and subsequent cardiac fibroblast activation.

**Figure 4:**
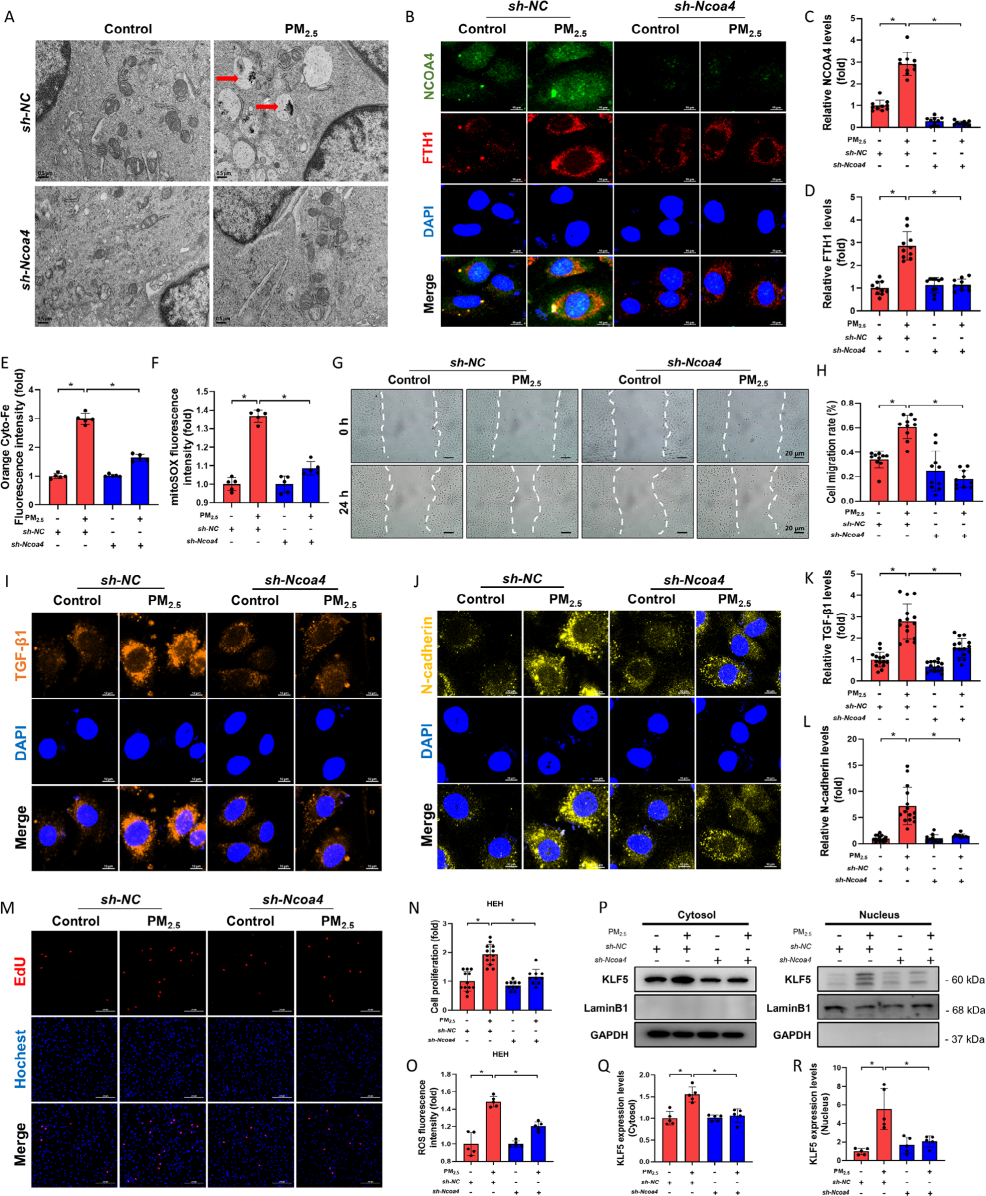
PM_2.5_ regulated iron homeostasis and EndMT through NCOA4. A) Representative TEM images of HAECs (Red arrows: autophagic lysosomes; Scale bar: 0.5 µm). B–D) Representative immunofluorescence images and semi‐quantitative analysis of NCOA4 and FTH1 proteins (scale bar: 10 µm). E, F) Analysis of intracellular Fe^2+^ and mitochondrial ROS by flow cytometry. G) Representative images of cell migration assay (scale bar: 20 µm). H) Semi‐quantitative analysis of cell migration rate. I, J) Representative immunofluorescence images of TGF‐β1 and N‐cadherin proteins (scale bar: 10 µm). K, L) Semi‐quantitative analysis of TGF‐β1 and N‐cadherin. M) Representative fluorescence images of EdU staining (scale bar: 50 µm). N) Semi‐quantitative analysis of the degree of HEH proliferation. O) Analysis of ROS content by flow cytometry. P) Representative Western blot pictures of KLF5 in cytosol and nucleus. Q, R) Semi‐quantitative analysis of KLF5 in cytosol and nucleus. n=5. All data were expressed as mean ± standard deviation. *p < 0.05.

To further explore the role of NCOA4 in PM_2.5_‐induced iron homeostatic imbalance, lentivirus‐mediated knockdown of the *Ncoa4* gene was performed in HAECs (Figure S7A, Supporting Information). TEM observations revealed that PM_2.5_ increased the number of autophagic lysosomes in the cells, while knockdown of *Ncoa4* significantly alleviated PM_2.5_‐induced autophagy (**Figure** **4**A). Immunofluorescence analysis showed that inhibition of NCOA4 expression attenuated the PM_2.5_‐induced increase in FTH1 expression (Figure 4B–D), suggesting that ferritinophagy was reduced. In addition, knockdown of NCOA4 attenuated the effects of PM_2.5_ on intracellular Fe^2+^ content and mtROS, while restoring MMP after PM_2.5_ exposure, indicating that reduced NCOA4 expression mitigated the dysregulation of intracellular iron homeostasis caused by PM_2.5_ (Figure 4E, F; Figure S7B, Supporting Information). Similarly, knockdown of *Ncoa4* significantly alleviated the upregulation of p62, LC3B‐II, MDA, and 4‐HNE expression induced by PM_2.5_ exposure (Figure S7C–H, Supporting Information), indicating that NCOA4 participates in the regulation of PM_2.5_‐induced ferritinophagy and iron homeostasis imbalance.

EndMT is known to enhance endothelial cell migration, and scratch assay results revealed that PM_2.5_ exposure promoted HAEC migration, an effect that was significantly blocked by suppressing NCOA4 expression (Figure 4G, H). Immunofluorescence assays further demonstrated that *Ncoa4* knockdown alleviated PM_2.5_‐induced upregulation of TGF‐β1 and N‐cadherin (Figure 4I–L). Furthermore, when the conditioned medium from four groups of HAECs was applied to HEH, conditioned medium from the exposure group induced HEH hyperproliferation, elevated ROS levels, and fibrotic phenotypic transformation. However, knockdown of *Ncoa4* in HAECs significantly alleviated the proliferative activation of HEH (Figure 4M–O; Figure S7I–K, Supporting Information). These results suggest that PM_2.5_ exposure promotes cardiac fibroblast proliferation by activating endothelial cell ferritinophagy. Interestingly, NCOA4 was also found to modulate the effect of PM_2.5_ on KLF5 expression. Moreover, PM_2.5_‐induced nuclear translocation of KLF5 was reduced following NCOA4 knockdown (Figure 4P–R), suggesting that PM_2.5_ may stimulate KLF5 nuclear translocation via NCOA4, thereby enabling KLF5 to regulate downstream fibrotic genes.

### 2.6 PM_2.5_ Evoked Transcription Factor KLF5 to Promote TGF‐β1 Transcription

**Figure 5:**
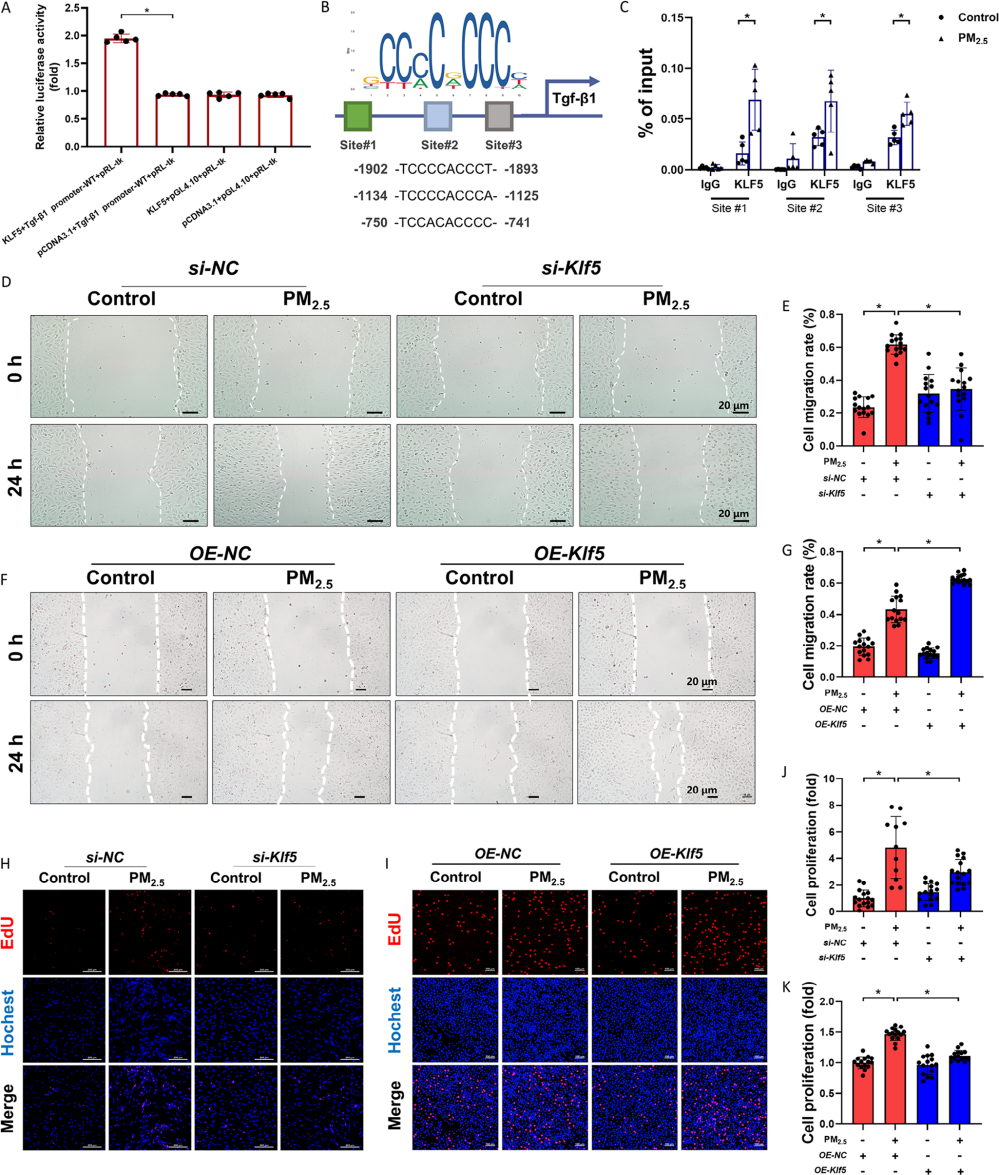
PM_2.5_ evoked transcription factor KLF5 to promote *Tgf‐β1* transcription. A) Dual luciferase reporter assay. B) Schematic of the KLF5‐binding site on human *Tgf‐β1* gene. C) KLF5 ChIP experiments (IgG as an internal control). D) Representative images of cell migration assay (scale bar: 20 µm). E) Semi‐quantitative analysis of cell migration rate. F) Representative images of cell migration assay (scale bar: 20 µm). G) Semi‐quantitative analysis of cell migration rate. H, I) Representative fluorescence images of EdU staining (scale bar: 20 µm). J, K) Semi‐quantitative analysis of the degree of HEH proliferation. n=5. All data were expressed as mean ± standard deviation. *p < 0.05.

The JASPAR database (https://jaspar.genereg.net) was used to identify potential KLF5‐binding sites on the *TGF‐β1* promoter region. The dual‐luciferase reporter assay showed that KLF5 could directly bind to the *TGF‐β1* promoter at the predicted sites (**Figure** **5**A). Analysis of the promoter region (−2000 to +1 bp) of human TGF‐β1 using JASPAR predicted three possible KLF5‐binding regions (Site 1, ‐1902 TCCCCACCCT ‐1893; Site 2, ‐1134 TCCCCACCCA ‐1125; Site 3, ‐750 TCCACACCCC ‐741) (Figure 5B). Chromatin immunoprecipitation ‐ quantitative polymerase chain reaction (ChIP‐qPCR) assay demonstrated that after PM_2.5_ exposure, KLF5 occupancy on the *TGF‐β1* promoter in endothelial cells was increased (Figure 5C). To further verify the role of KLF5 in TGF‐β1 regulation and fibrotic signaling, both *Klf5* knockdown and *Klf5* overexpression were performed in HAECs. *Klf5* siRNA significantly reduced PM_2.5_‐induced HAEC migration in scratch assays and prevented the upregulation of TGF‐β1 and N‐cadherin (Figure 5D, E; Figure S8A–E, Supporting Information). In co‐culture, conditioned medium from *Klf5*‐silenced HAECs failed to stimulate HEH proliferation, ROS accumulation, and myofibroblast phenotypic transformation (Figure 5H and J; Figure S8F–I, Supporting Information). Conversely, KLF5 overexpression in PM_2.5_‐exposed HAECs further enhanced cell migration, increased TGF‐β1 and N‐cadherin expression, and amplified HEH proliferation in co‐culture (Figure 5F, G, I, and K; Figure S8J–N, Supporting Information). Taken together, these findings demonstrated that PM_2.5_‐induced activation and nuclear recruitment of KLF5 could directly upregulate the *TGF‐β1* transcription via specific promoter binding, thereby driving EndMT and paracrine fibrotic signaling.

### 2.7 PM_2.5_ Triggered Cardiac Dysfunction and Pathological Damage in Mice via NCOA4

**Figure 6:**
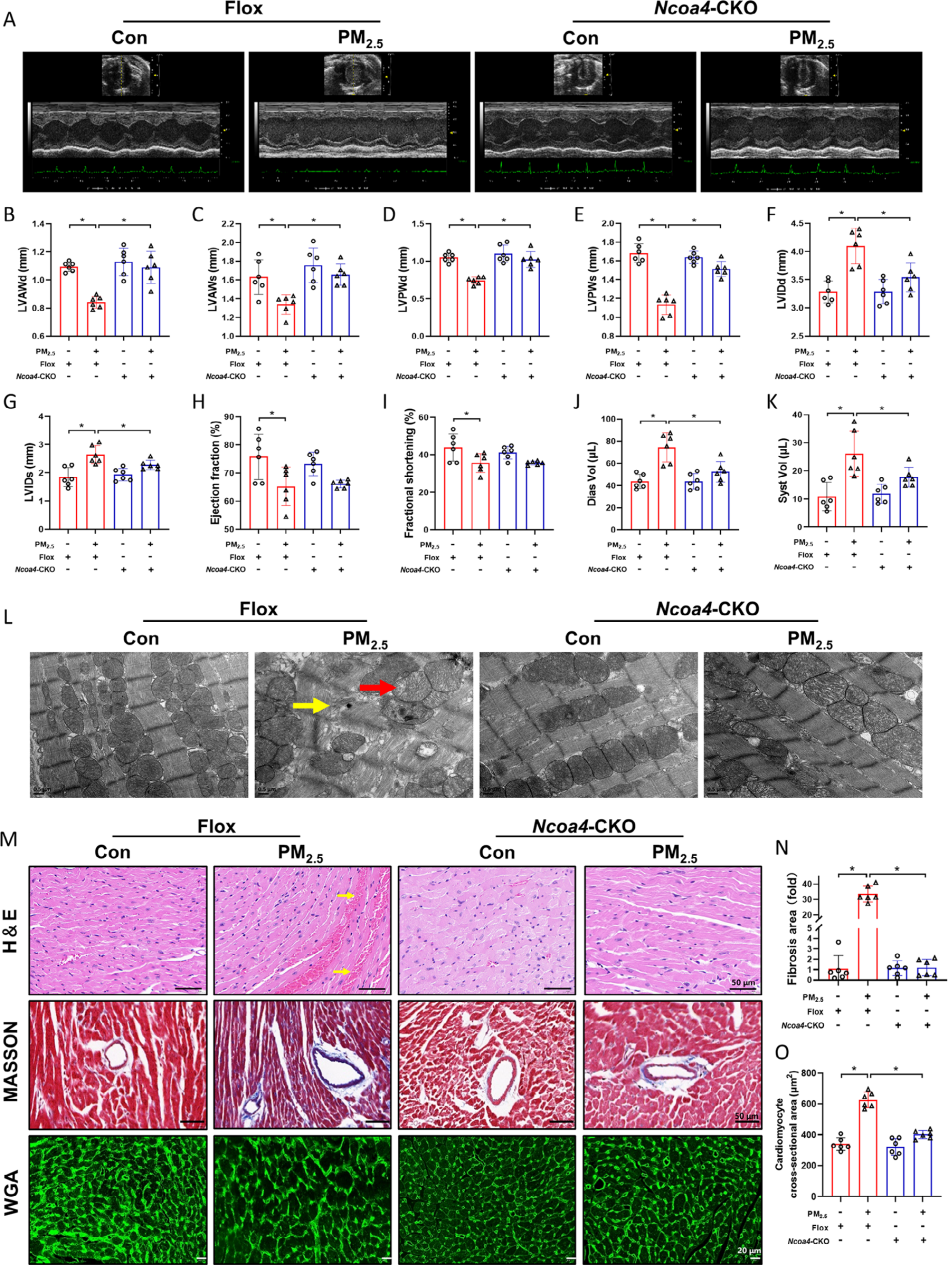
PM_2.5_ triggered cardiac dysfunction and pathological damage in mice via the activation of NCOA4. A) M‐mode echocardiography. B) Left ventricular anterior wall thickness at diastole. C) Left ventricular anterior wall thickness at systole. D) Left ventricular posterior wall thickness at diastole. E) Left ventricular posterior wall thickness at systole. F) Left ventricular internal diameter at diastole. G) Left ventricular internal diameter at systole. H) Ejection fraction. I) Fractional shortening. J) Diastolic volume. K) Systolic volume. L) Representative TEM images of myocardial tissue (Yellow arrows: disturbed arrangement of myocardial fibers; Red arrows: swollen mitochondria; Scale bar: 0.5 µm). M) Representative images of H&E staining, MASSON staining and WGA staining in heart sections. N) Semi‐quantitative analysis of fibrosis area. O) Semi‐quantitative analysis of cardiomyocyte cross‐sectional area. n=6. All data were expressed as mean ± standard deviation. *p < 0.05.

In vivo experiments using transgenic mice were conducted to investigate the effects of PM_2.5_ on cardiac structure and function, with an average daily PM_2.5_ exposure concentration of 146 µg m^−3^ (Figure S9A–C, Supporting Information). Doppler ultrasound examination showed that LVAWd, LVAWs, LVPWd, and LVPWs were decreased after PM_2.5_ exposure, while LVIDd and LVIDs were increased (**Figure** **6**A–G), suggesting that PM_2.5_ increased myocardial stiffness in the left ventricle (LV), leading to both diastolic and systolic dysfunction. Moreover, ejection fraction and fractional shortening were significantly reduced, while diastolic and systolic volumes were significantly elevated after PM_2.5_ exposure, indicating impaired relaxation of the LV and reduced contractile capacity of cardiomyocytes in mice (Figure 6H–K). These adverse effects were ameliorated by conditional knockout of the *Ncoa4* gene in endothelial cells. TEM revealed disturbed arrangement of myofibers in myocardial tissue, swollen mitochondria, and disrupted mitochondrial cristae in cardiomyocytes (Figure 6L). H&E staining of heart tissue showed that PM_2.5_ exposure induced pathological changes, including increased cytoplasmic vacuolization, myofibrillar disruption, and large extravascular erythrocyte accumulation between cardiomyocytes. Masson staining demonstrated that PM_2.5_ exposure induced cardiac fibrosis, predominantly perivascular fibrosis in mice. The WGA staining revealed that PM_2.5_ exposure enlarged the cross‐sectional area of cardiomyocytes, which was regarded as a sign of cardiac hypertrophy. Notably, PM_2.5_‐induced cardiotoxicity was attenuated in *Ncoa4^fl/fl,Cdh5‐cre^* mice (Figure 6M–O).

### 2.8 PM_2.5_ Induced Cardiac Fibrosis by Activating Ferritinophagy

**Figure 7:**
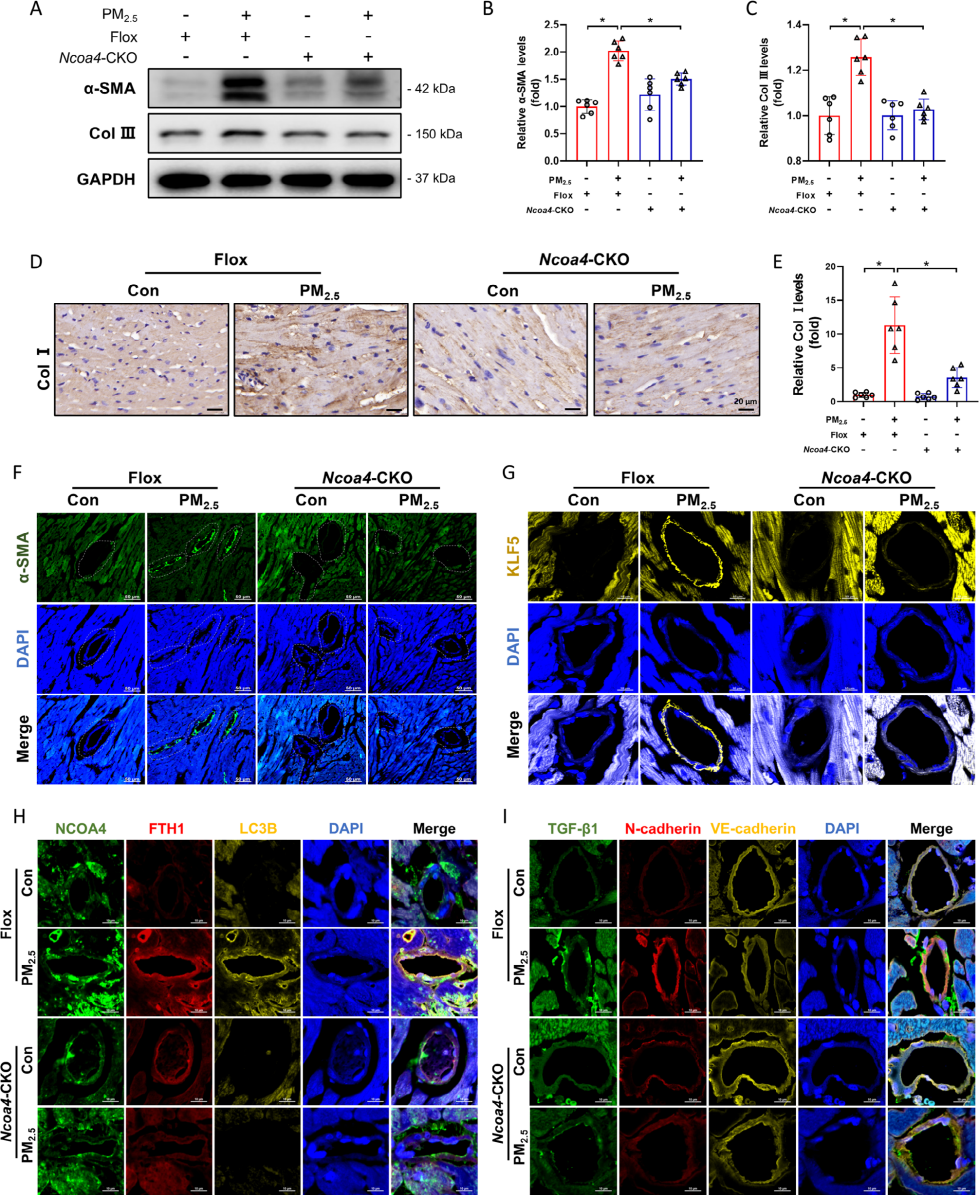
PM_2.5_ induced cardiac fibrosis by activation of endothelial cell ferritinophagy. A) Representative Western blot pictures. B, C) Semi‐quantitative analysis of α‐SMA and Collagen III. D, E) Representative immunohistochemical images and semi‐quantitative analysis of Collagen I. F) Representative immunofluorescence images of α‐SMA. G) Representative immunofluorescence images of KLF5. H) Representative immunofluorescence images of NCOA4, FTH1 and LC3B. I) Representative immunofluorescence images of TGF‐β1, N‐cadherin and VE‐cadherin. n=6. All data were expressed as mean ± standard deviation. *p < 0.05.

Compared with *Ncoa4^fl/fl^* mice, PM_2.5_ induced cardiac fibrosis was significantly attenuated in *Ncoa4^fl/fl,Cdh5‐cre^* mice. Moreover, Western blot and immunohistochemistry assays showed that PM_2.5_‐induced upregulation of α‐SMA, Col I, and Col III expression was also ameliorated in *Ncoa4^fl/fl,Cdh5‐cre^* mice (**Figure** **7**A–E). The localization of α‐SMA and KLF5 in mouse hearts also suggested that PM_2.5_ induced EndMT, which was mitigated by ferritinophagy inhibition (Figure 7F–G). In addition, Fe^2+^ content analysis demonstrated that PM_2.5_ caused Fe^2+^ accumulation in endothelial cells of cardiac blood vessels (Figure S9D, Supporting Information). PM_2.5_ upregulated the expression of NCOA4, FTH1, LC3B, N‐cadherin, and TGF‐β1, while downregulating VE‐cadherin in cardiac endothelial cells. These results indicated that PM_2.5_ induced ferritinophagy and EndMT in cardiac endothelial cells (Figure 7H, I). Conditional knockout of the *Ncoa4* gene in endothelial cells alleviated these changes. Taken together, these findings suggested that PM_2.5_ induced EndMT in mouse hearts via endothelial cell ferritinophagy, ultimately leading to cardiac fibrosis.

## 3 Discussion

The burden of cardiovascular disease caused by air pollution, particularly by the major pollutant PM_2.5_, is a significant global public health concern.^[^ ^2^, ^23^, ^24^, ^25^ ^]^ A growing number of studies have reported that metallic elements such as iron, copper and cadmium in PM_2.5_ are associated with cardiovascular dysfunction.^[^ ^24^, ^25^ ^]^ In this study, we revealed for the first time the specific role of PM_2.5_‐derived Fe‐containing particles in mediating ferritinophagy using spICP‐TOF‐MS and molecular dynamics simulations, and demonstrated that exogenous iron was a major initiator of PM_2.5_‐induced disturbances in iron homeostasis. This dysregulation promoted EndMT via the NCOA4/KLF5 pathway, thereby contributing to PM_2.5_‐induced cardiac fibrosis.

Previous studies have shown that exogenous particulate matter inhaled through respiration can penetrate the air‐blood barrier and enter the circulatory system, where it is distributed to different organs via blood circulation.^[^ ^26^ ^]^ It has been demonstrated that Fe‐containing air pollution particles can invade the human myocardium and cause myocardial iron overload and oxidative damage, thereby exacerbating cardiovascular injury.^[^ ^11^, ^27^ ^]^ These studies provided direct evidence for the entry of PM_2.5_ into the human circulatory system and the associated health risks. In this study, we highlighted that Fe‐containing particles from PM_2.5_ were more likely to enter the bloodstream than other metal‐containing particles and can accumulate in heart tissue. More importantly, Fe‐containing particles in heart tissue exhibited a higher Fe content and smaller sizes compared with those in lung and serum (Figure 1). In addition, Fe‐containing particles observed in heart tissue were significantly larger than ferritin‐derived magnetite nanoparticles in in vivo controls, consistent with previous findings in human samples.^[^ ^9^ ^]^ These spherical Fe‐containing particles resemble those derived from combustion activities, such as coal combustion, which have also been observed in the human heart, brain, serum, and pleural effusions.^[^ ^9^, ^28^ ^]^ Furthermore, Fe‐containing particles with higher abundance and smaller sizes may generate greater ROS production and persist longer in the heart compared with other highly perfused organs, thereby posing potentially greater risks to the cardiovascular health.^[^ ^27^, ^29^, ^30^ ^]^

PM_2.5_ have been extensively studied for its role in cardiac injury, including oxidative stress and inflammation. However, our findings uniquely identified Fe‐containing particles as a critical component driving dysregulated ferritinophagy. Previous studies have reported increased systemic iron levels after PM_2.5_ exposure,^[^ ^31^ ^]^ but few have directly linked this to cardiac fibrosis through ferritinophagy. Fe‐containing particles may induce a more pronounced disruption of cellular iron regulation, leading to increased intracellular Fe^2+^ compared with other PM_2.5_ components. Elevated Fe^2+^ can trigger a cascade of events, including ferritinophagy.^[^ ^32^ ^]^ Ferritinophagy is a process in which NCOA4 functions as a cargo receptor for ferritin, mediating its delivery to autophagic vesicles and promoting ferritin‐targeted lysosomal degradation to release iron ions.^[^ ^14^, ^15^, ^33^ ^]^ Notably, PM_2.5_ derived Fe‐containing particles (Fe_3_O_4_) were identified as key contributors capable of directly interacting with NCOA4. Our previous work and other studies have shown that inhaled PM_2.5_‐derived Fe‐containing particles translocate from the lungs into the circulation and accumulate in tissues predominantly as magnetite (Fe_3_O_4_), ^[^ ^34^, ^35^ ^]^ making Fe_3_O_4_ the biologically relevant form for our molecular simulations. Structural dynamics analysis demonstrated that NCOA4 underwent conformational changes upon binding to Fe_3_O_4_, adopting a more compact conformation with decreased solvent‐exposed surface area, while the Fe_3_O_4_ nanoparticles remained highly structurally stable (Figure 2).

To complement these computational findings, we extracted the magnetite‐enriched fraction from PM_2.5_ and exposed endothelial cells to these particles. This “Fe‐containing” fraction was obtained using our established cyclic magnetic extraction protocol,^[^ ^22^, ^36^ ^]^ which enriches particles with sufficient magnetic response. Comprehensive TEM characterization consistently identified magnetite as the predominant phase in this fraction, despite the possible coexistence of minor magnetic minerals (e.g., hausmannite). ^[^ ^34^, ^37^ ^]^ These PM_2.5_‐derived Fe‐containing particles caused a significant upregulation of NCOA4 protein levels in endothelial cells in vitro, closely mirroring the predicted docking interface and confirming a biologically meaningful interaction. These findings supported the hypothesis that Fe‐containing particles affect the physiological function of NCOA4 by altering its protein structure, thereby promoting ferritinophagy and disturbing iron homeostasis. Our results revealed that PM_2.5_ exposure significantly altered iron homeostasis in endothelial cells by inducing ferritinophagy. The upregulation of NCOA4, together with increased intracellular Fe^2+^ and mitochondrial ROS levels, implicated ferritinophagy as a key driver of mitochondrial damage and cellular dysfunction (Figure 3). Collectively, these findings provided strong evidence that Fe‐containing particles from PM_2.5_ were not merely passive environmental toxins but active mediators of iron metabolism disruption and cardiovascular injury.

EndMT is a distinct form of endothelial cell injury associated with numerous pathophysiological processes, including cardiac fibrosis, atherosclerosis, and pulmonary arterial hypertension.^[^ ^38^, ^39^, ^40^, ^41^ ^]^ Lineage‐tracing studies in mouse models of pressure overload and cardiac injury have shown that up to 30–35 % of fibroblasts in fibrotic myocardium originate from endothelial cells via EndMT.^[^ ^17^ ^]^ In this study, we found that PM_2.5_ promoted EndMT, suggesting that PM_2.5_ may induce cardiac fibrosis by first triggering phenotypic transformation of endothelial cells. Currently, there is a limited understanding of how PM_2.5_ regulates endothelial cells to induce cardiac fibrosis, ^[^ ^42^ ^]^ and the interplay between ferritinophagy and EndMT remains relatively unexplored. Our study demonstrated that PM_2.5_ induced abnormal ferritinophagy leading to EndMT, which could represent a potential mechanism for PM_2.5_‐induced cardiac fibrosis (Figure 4). Excessive proliferation of cardiac fibroblasts and abnormal deposition of extracellular matrix components are the primary drivers of the cardiac fibrotic process.^[^ ^43^ ^]^ Our previous reports showed that PM_2.5_ upregulated ROS levels in cardiac fibroblasts, inducing their phenotypic transformation into myofibroblasts and promoting the progression of cardiac fibrosis.^[^ ^44^ ^]^ In this study, we similarly found that PM_2.5_ activated cardiac myofibroblasts by inducing endothelial cell ferritinophagy and EndMT, resulting in enhanced proliferation and ROS overproduction.

Notably, recent reports have indicated that iron overload accelerates EndMT in atherosclerosis, a finding consistent with our observations in PM_2.5_‐exposed models. However, whereas their study attributed this effect primarily to microenvironmental crosstalk, we identified KLF5 as a key regulator, suggesting a more precise regulatory network.^[^ ^16^ ^]^ KLF5 has been proposed to interact with NCOA4 based on existing evidence of their biological functions. NCOA4‐mediated ferritinophagy releases free iron, leading to Fenton reactions and lipid peroxidation in cells.^[^ ^45^ ^]^ KLF5 is known to be responsive to oxidative and fibrogenic stimuli and to regulate a broad transcriptional network involved in extracellular matrix production and fibroblast activation.^[^ ^46^, ^47^ ^]^ Based on this evidence, we hypothesized that PM_2.5_ act on NCOA4 to induce KLF5 activation. In the present study, exposure to Fe‐containing particles from PM_2.5_ markedly increased in NCOA4 expression, which was accompanied by upregulation of KLF5. Using lentiviral transduction, we observed that suppressing NCOA4 not only reduced lipid peroxidation but also prevented the induction of KLF5. This suggested that iron overload and oxidative stress caused by NCOA4 activation might serve as triggers for KLF5 induction. Taken together, these findings supported a coherent and biologically plausible pathway: NCOA4‐mediated Fe^2+^ release and oxidative stress lie upstream of KLF5 induction, positioning KLF5 as a downstream effector in PM_2.5_‐induced cardiac fibrosis. We further verified that KLF5 binds to the *TGF‐β1* promoter region by dual‐luciferase reporter assay, which may represent an important mechanism by which PM_2.5_ promote the EndMT process. The above results indicated that KLF5 was a key regulator in promoting fibrosis, which may help elucidate PM_2.5_‐induced signal transduction in cardiac fibrosis and, in turn, served as a potential biomarker in the future (Figure 5). Consistent with the results of the in vitro experiments, we found that PM_2.5_ induced cardiac dysfunction and fibrosis in mice, characterized by predominant perivascular fibrosis (Figure 6). Moreover, ferritinophagy and EndMT were also observed in cardiac vessels (Figure 7). Our results identified endothelial cell regulation of cardiac fibroblasts through ferritinophagy and EndMT as a novel mechanism of PM_2.5_‐induced cardiac fibrosis. In the future, investigating interactions among endothelial cells, cardiac fibroblasts, and cardiomyocytes may provide a promising strategy for studying the cardiotoxicity of PM_2.5_.

**Figure 8:**
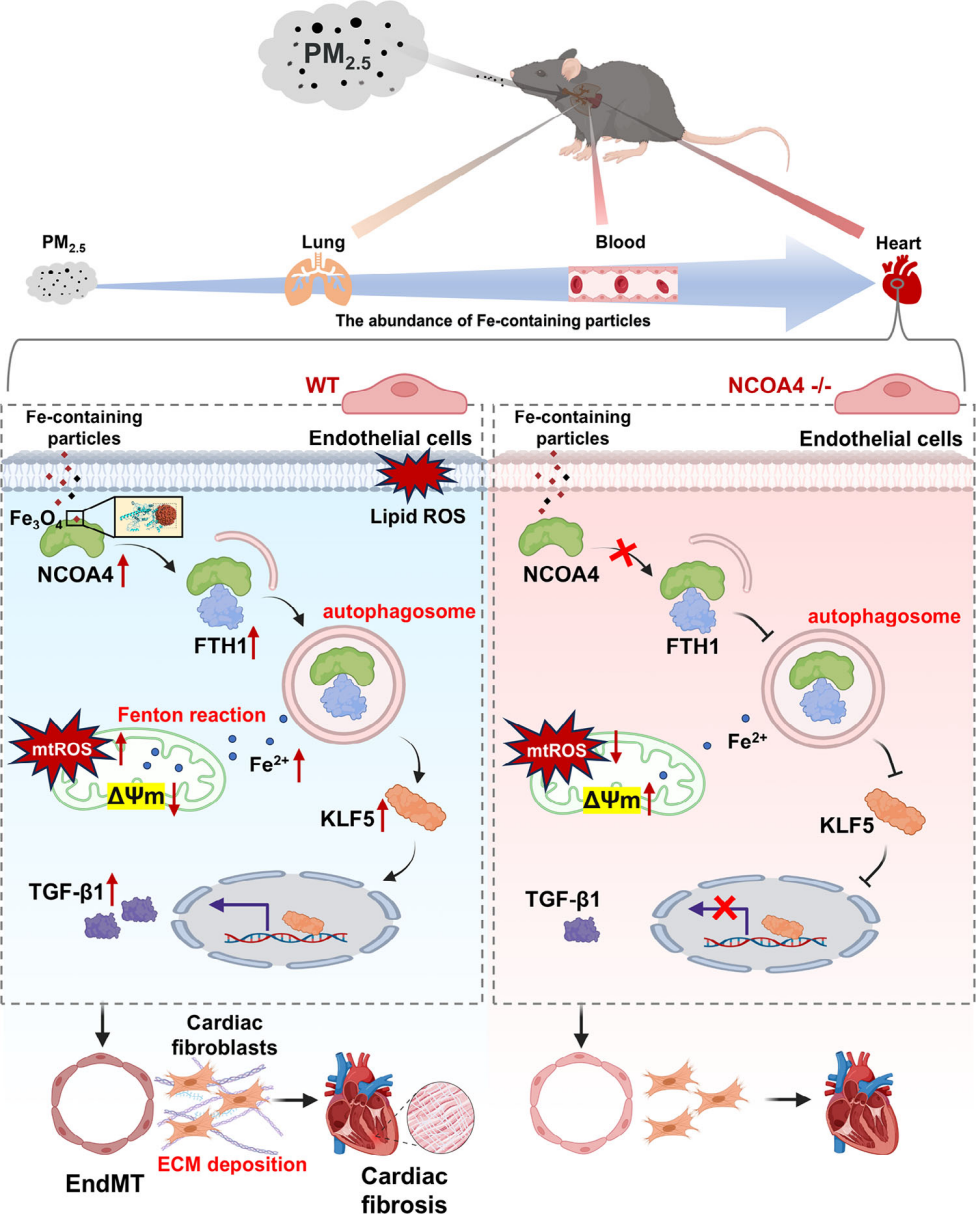
The schematic of PM_2.5_‐promote the EndMT and cardiac fibrosis by triggering iron homeostatic imbalance. PM_2.5_ derived Fe‐containing particles accumulate in endothelial cells and interact with NCOA4, promoting ferritinophagy. This process leads to the degradation of FTH1, releasing Fe^2+^ and disrupting intracellular iron homeostasis. Elevated iron levels trigger mitochondrial dysfunction, increased ROS production, and lipid peroxidation. Furthermore, PM_2.5_‐induced ferritinophagy stimulates EndMT via the NCOA4/KLF5 signaling pathway. Endothelial cells secrete increased levels of TGF‐β1, which influences the phenotype transformation of cardiac fibroblasts. These fibroblasts proliferate and deposit extracellular matrix, ultimately driving cardiac fibrosis and dysfunction.

To the best of our knowledge, no prior study has reported a role for the NCOA4/KLF5 signaling axis in organ fibrosis, underscoring the novelty of our findings. Although studies have shown that NCOA4 drives ferroptosis in fibrosis and KLF5 regulates fibrotic signaling, their interaction has never been investigated. For instance, elevated NCOA4 expression has been demonstrated in fibroblastic areas of idiopathic pulmonary fibrosis, where NCOA4‐driven ferritin degradation increases intracellular iron and catalyzes Fenton reactions, leading to lipid peroxidation, ferroptotic injury of epithelial cells, and fibrotic remodeling.^[^ ^48^, ^49^ ^]^ Inhibition of iron overload has been shown to attenuate fibrosis in animal models of pulmonary injury.^[^ ^50^, ^51^ ^]^ Similarly, NCOA4‐mediated iron recycling influences hepatocyte behavior during liver fibrosis.^[^ ^52^ ^]^ Moreover, KLF5 regulates extracellular matrix production, fibroblast activation, and inflammatory signaling in multiple tissues including the lung, kidney, and liver.^[^ ^53^, ^54^ ^]^ Our data demonstrated that PM_2.5_‐derived Fe‐containing particles triggered NCOA4‐dependent ferritinophagy and lipid peroxidation, which in turn induced KLF5 expression and led to cardiac fibrosis (Figure 8). This cascade may also play a critical role in fibrotic diseases across multiple organ systems.

Furthermore, exposure to Fe‐rich environmental agents such as diesel particulates and welding fumes has been shown to exacerbate pulmonary and hepatic fibrosis via oxidative stress and ferroptosis pathways.^[^ ^55^, ^56^, ^57^ ^]^ These findings reinforced the possibility that the NCOA4/KLF5 pathway may represent a generalizable mechanism of iron‐driven fibrotic disease induced by environmental pollutants. Therefore, future research should investigate this signaling pathway in lung, liver, and kidney fibrosis models, as well as in contexts involving diverse Fe‐containing exposures.

## 4 Conclusion

In summary, our study demonstrates that PM_2.5_‐derived Fe‐containing particles can enhance ferritinophagy and promote iron overload in endothelial cells. Molecular docking and dynamics simulations confirmed that magnetite particles are key contributors, capable of directly interacting with NCOA4. Additionally, PM_2.5_ exposure further trigger the EndMT via NCOA4/KLF5 signaling pathway, ultimately leading to cardiac fibrosis and dysfunction. Our data primarily justify the role of exogenous Fe‐containing particles in PM_2.5_‐induced disturbances of iron homeostasis, highlighting that targeting ferritinophagy and EndMT may represent a promising strategy to mitigate the burden of PM_2.5_‐associated cardiovascular disease.

## 5 Experimental Section

### PM_2.5_ Samples Preparation

PM_2.5_ samples were collected from the Capital Medical University using a high‐flow atmospheric particulate sampler (TH‐1000C II, Wuhan Tianhong, China). Subsequently, the collected PM_2.5_ were eluted, lyophilized, and then sterilized with UV light.

### Experimental Animals and Treatments

All animals in this study were approved by the Experimental Animal Welfare Committee of Capital Medical University, and the ethic number was AEEI‐2023‐258. All mice were on a C57BL/6J background. CRISPR/Cas9 technology was used to design and construct specific sgRNA and ssDNA to obtain F0 generation positive mice *Ncoa4^fl/+^* by high‐throughput electro transfer of fertilized eggs, which meant that a loxp sequence was inserted into Intron 1 and Intron 6, respectively, to obtain F0 generation mice. *Ncoa4^fl/+^* mice were obtained and self‐crossed to obtain *Ncoa4^fl/fl^* mice. Then hybridization with *Cdh5‐Cre* transgenic mice was performed to obtain vascular endothelial cell‐specific knockout heterozygous mice (*Ncoa4^fl/+,Cdh5‐cre^*) followed by self‐crossing to obtain vascular endothelial tissue‐specific knockout pure mice (*Ncoa4^fl/fl,Cdh5‐cre^*). Mice of both genotypes (*Ncoa4^fl/fl^* and *Ncoa4^fl/fl,Cdh5‐cre^*) were randomly divided into the control group or the PM_2.5_ exposure group, and littermates were used as controls in all experiments. Mice in the control group were placed in a clean air chamber (1 µg m^−3^) equipped with a high‐efficiency particulate air filter. Mice in the PM_2.5_ exposure group received high concentrations of PM_2.5_ ambient air through a PM_2.5_ concentration enrichment system (Beijing Huironghe Technology Co., Ltd, Beijing, China) in the whole‐body exposure chamber. An aerosol monitor (TSI Instrument Co., Ltd, Minneapolis, USA) was used to monitor PM_2.5_ concentrations in the exposure chamber. The exposure period was 6 hours per day for 90 consecutive days.

### Measurement of Elemental Concentrations

The elemental concentrations (Na, Mg, Al, K, Ca, Ti, V, Cr, Mn, Fe, Co, Ni, Cu, Zn, Sr, Zr, Nb, Sn, Sb, Ba, W, and Pb) of the magnetic fine particles from PM_2.5_ and tissues were determined by inductively coupled plasma mass spectrometer (PerkinElmer NexION 350D, United States). The samples were digested with a mixture of HNO_3_‐HF‐HClO_4_ using a microwave digestion apparatus (WX‐8000, PreeKem Scientific Instruments Co., Ltd., China). In brief, 4 mL HNO_3_, 0.5 mL HF, and 0.5 mL HClO_4_ (electronic grade) were added to the sample, and then the mixture was irradiated at 220 °C for 30 min. After microwave digestion, concentrate the digests to 200 µL by acid evaporation, which were transferred to a 10 mL polyethylene centrifuge tube and then diluted to 5 mL with ultrapure water for ICP‐MS analysis. The blank control sample was also analyzed following the same procedures to subtract the background.

### Sample Pre‐Treatment

For PM_2.5_ samples collected on quartz filters, 1.52 cm^2^ of each filter was taken and mixed, and magnetite particles were extracted after digestion with KOH with reference to the previous method.^[^ ^22^ ^]^ Briefly, the quartz filters were digested in 5 M KOH solution by sonication followed by heating at 90 °C for 12 h. The digested solution was then introduced into the CME unit to extract the magnetic particles, and 2 m acetic acid was added to the extract to remove impurities in order to obtain the magnetite particles.

The extraction method of magnetite particles from lung and heart tissues was described in detail elsewhere.^[^ ^36^ ^]^ The brief description was as follows: Mouse tissues were digested for 24 h at room temperature with 20% TMAH at a ratio of 1: 20 (the tissues: TMAH, m/m). The tissue digestion solutions were then treated by the CME method with an additional washing step with 2 m acetic acid after TMAH digestion. In addition, pure alcohol and ultrapure water were used to wash the magnetite matter after acid purification before spICP‐TOF‐MS analysis. Mouse serums were washed three times with ultrapure water by using a centrifugal filtration device containing a porous cellulose membrane (MWCO:3 KD).

### spICP‐TOF‐MS Analysis

Each sample was introduced into spICP‐TOF‐MS for individual particle elemental composition analysis after sufficient dilution with ultrapure water. The instrument operating conditions were summarized in Table S1 (Supporting Information). A standard solution of 50 nm Au‐nanoparticles was used to calculate the transmission efficiency. Dissolved calibration standards were prepared from a mixed multielement ICP certified reference standard (0, 0.05, 0.1, 0.5, 1, 5, 10 µg L^−1^) to determine the elemental specific mass responses of particles. All samples and blank samples were analyzed in triplicate. The mass detection limits of elements were presented in Table S2 (Supporting Information). A satisfactory recovery of 73% was obtained for spiked Au nanoparticles. Detailed methods are provided in the Supporting Information.

## Conflict of Interest

The authors declare no conflict of interest.

## Supporting information



Supporting Information

Supplementary Video S1

## Data Availability

The data that support the findings of this study are available from the corresponding author upon reasonable request.
