# Supplementary material for: PM2 .5 Induce Endothelial‐Mesenchymal Transition and Cardiac Fibrosis via the NCOA4‐Mediated Ferritinophagy
Source: Adv Sci (Weinh). 2025 Sep 15;12(45):e07536. doi: 10.1002/advs.202507536 (PMC12677608; doi:10.1002/advs.202507536)
Supplement: Supplementary file 1 — Supporting Information [file ADVS-12-e07536-s001.docx]

**Supplementary materials**

**PM_2.5_ Induce Endothelial-Mesenchymal Transition and Cardiac Fibrosis via the NCOA4-Mediated Ferritinophagy**

Qinglin Sun^1,4,5#^, Mengyuan Wang^2,#^, Lin Liu^3^, Ruiyang Ding^1,4,5^, Kanglin Yan^1,4,5^, Shiqian Liu^1,4,5^, Xiaoke Ren^1,4,5^, Qing Xu^6^, Zhiwei Sun^1,4,5^, Qian Liu^3,^*, Yi Yang^2,^*, Junchao Duan^1,4,5^*

*^1^Department of Toxicology and Sanitary Chemistry, School of Public Health, Capital Medical University, Beijing 100069, China*

*^2^Key Laboratory of Geographic Information Science of the Ministry of Education, School of Geographic Sciences, East China Normal University, Shanghai 200241, China.*

*^3^State Key Laboratory of Environmental Chemistry and Ecotoxicology, Research Center for Eco-Environmental Sciences, Chinese Academy of Sciences, Beijing 100085, China.*

*^4^Laboratory for Clinical Medicine, Capital Medical University, Beijing 100069, China*

*^5^Beijing Key Laboratory of Environment and Aging, Capital Medical University, Beijing 100069, China*

*^6^Core Facilities for Electrophysiology, Core Facilities Center, Capital Medical University, Beijing 100069, China*

# These two authors contributed equally to the work.

* Corresponding authors

**Supplementary figures**


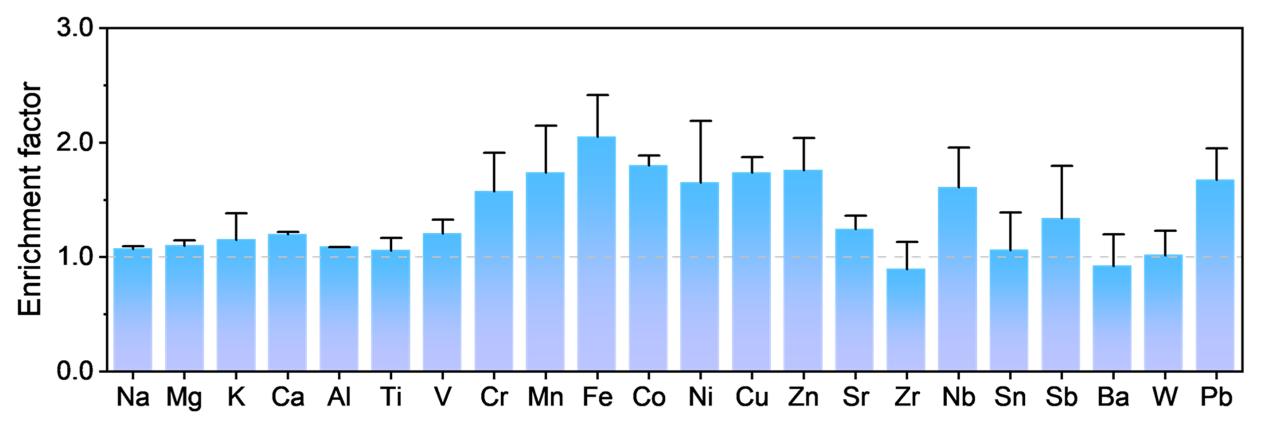


**Figure S1. Enrichment of metal-containing particles in mice tissues.** Enrichment factors of the 22 metal concentrations (ng/mL) in serum compared to the controls. n=8. All data were expressed as mean ± standard deviation.


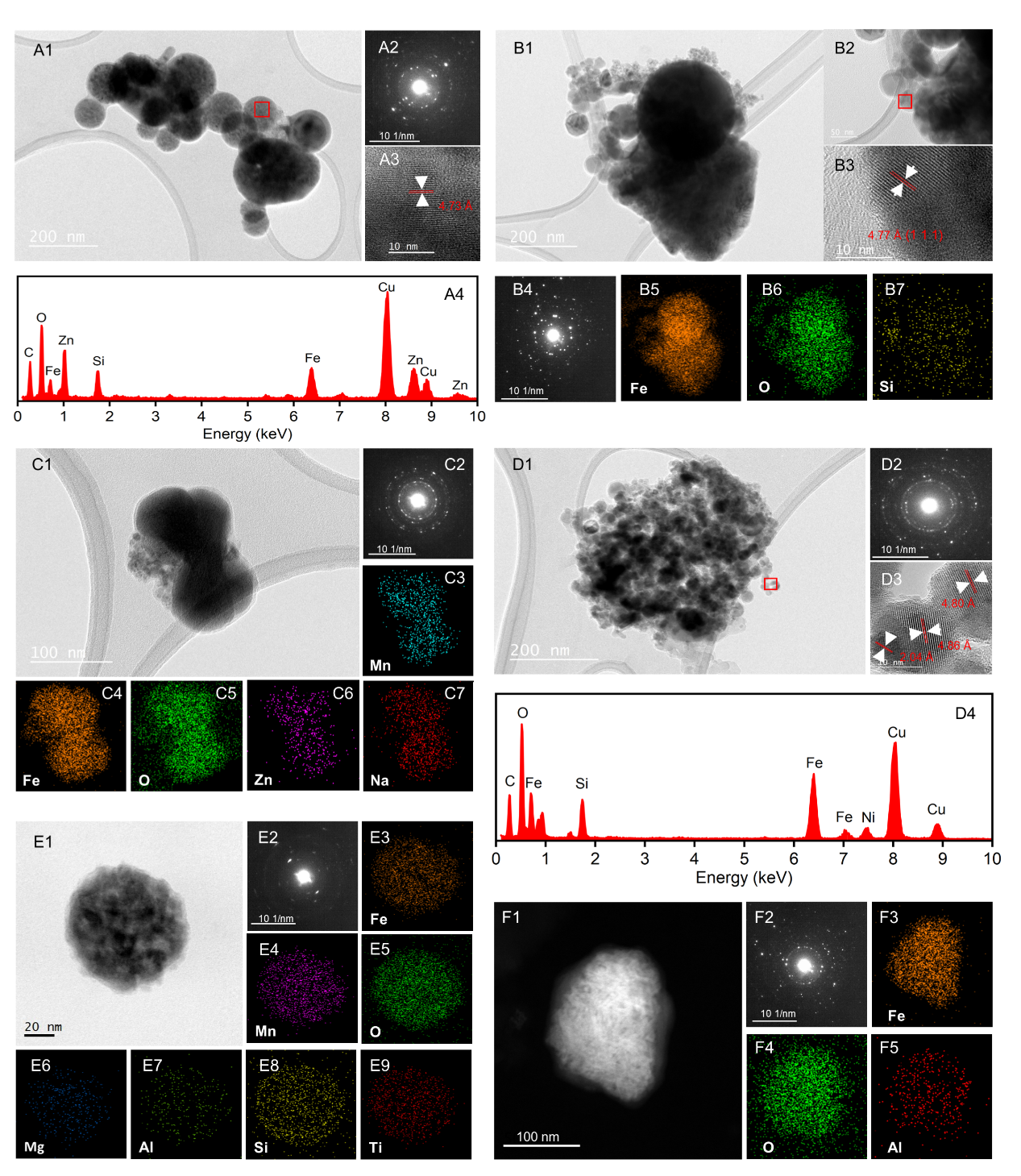


**Figure S2.** **Structural fingerprints of typical Fe-containing particles extracted from PM_2.5_, lung and serum.** (A1) TEM image of magnetite particles in PM_2.5_. (A2) Fast Fourier Transform (FFT) image of the red square marked magnetite particle in (A1), and (A3) high-resolution TEM image of the red square marked area. (A4) Energy dispersive X-ray (EDX) spectrum of magnetite particles in (A1). (B1) and (B2) TEM image of magnetite particles in PM_2.5_. (B3). High-resolution TEM image of the red square marked area in (B2). (B4) FFT image of the selected magnetite particles in (B2). (B5-7) EDX mapping of the selected particles in (B1). (C1) TEM image of hematite particle in lung tissue. (C2) FFT image of the particle. (C3-7) EDX mapping of the hematite particle in (C1). (D1) TEM image of nano-sized magnetite particles aggregates in lung tissue. (D2) FFT image of the aggregate. (D3) High-resolution TEM image of the red square marked area in (D1). (D4) EDX spectrum of the aggregate. (E1) Scanning transmission electron microscopy (STEM) image of nano-sized ilmenite particles from serum. (E2) FFT image of the particle. (E3-9) EDX mapping of the ilmenite particles in (E1). (F1) STEM image of nano-sized magnetite particles from serum. (F2) FFT image of the particle. (F3-5) EDX mapping of the magnetite particles in (F1). n=8.


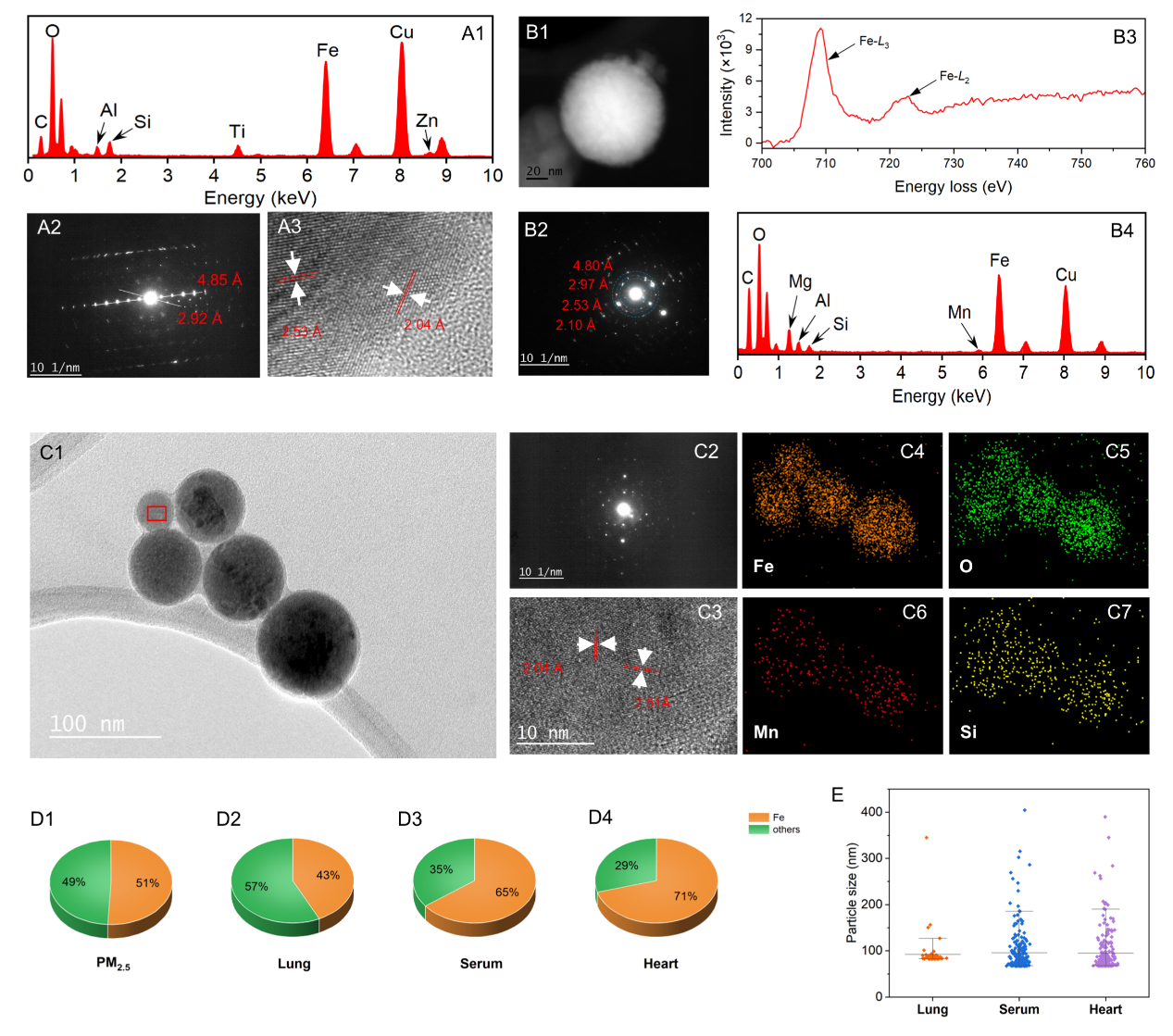


**Figure S3.** **Structural fingerprints of magnetite particles extracted from heart tissues.** (A1) EDX spectrum for the magnetite particles in Fig.1 (D1). (A2) FFT image of the magnetite particles in Fig.1 (D1) and (A3) high-resolution TEM image of the yellow square marked area. (B1) STEM image of nano-sized magnetite particles from heart and it’s (B2) FFT image, (B3) EELS and (B4) EDX spectrum. (C1) TEM image of nano-sized magnetite particles in heart tissue. (C2) FFT image of the red square marked area in (C1). (C3) High-resolution TEM image of the red square marked area in (C1). (C4-7) EDX mapping for the selected particles in (C1). (D1-4) Variations in the mass fractions of Fe among 22 metals in Fe-containing particles in PM_2.5_, lung, serum and heart. (E) Particle size of Fe-containing particles in control mice lung, serum and heart tissues. n=8.


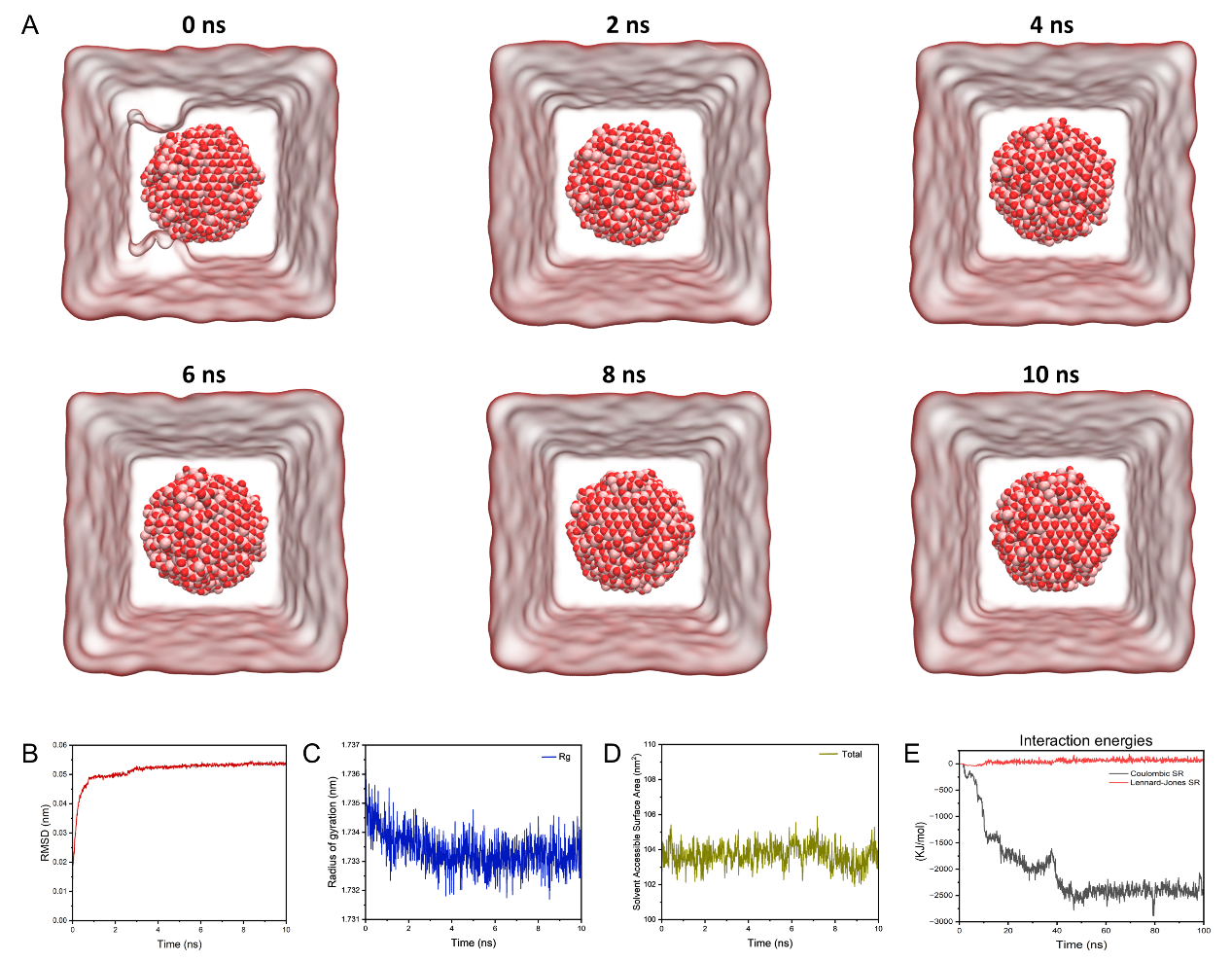


**Figure S4. Conformational analysis of Fe_3_O_4_ and interaction energy with NCOA4.** (A) Conformational evolutionary behavior of Fe_3_O_4_ nanoparticles in aqueous solution. (B) Root mean square deviation (RMSD). (C) Radius of gyration (Rg). (D) Solvent-accessible surface area (SASA). (E) The electrostatic interaction energy (Coul-SR) and the van der Waals interaction energy (LJ-SR).


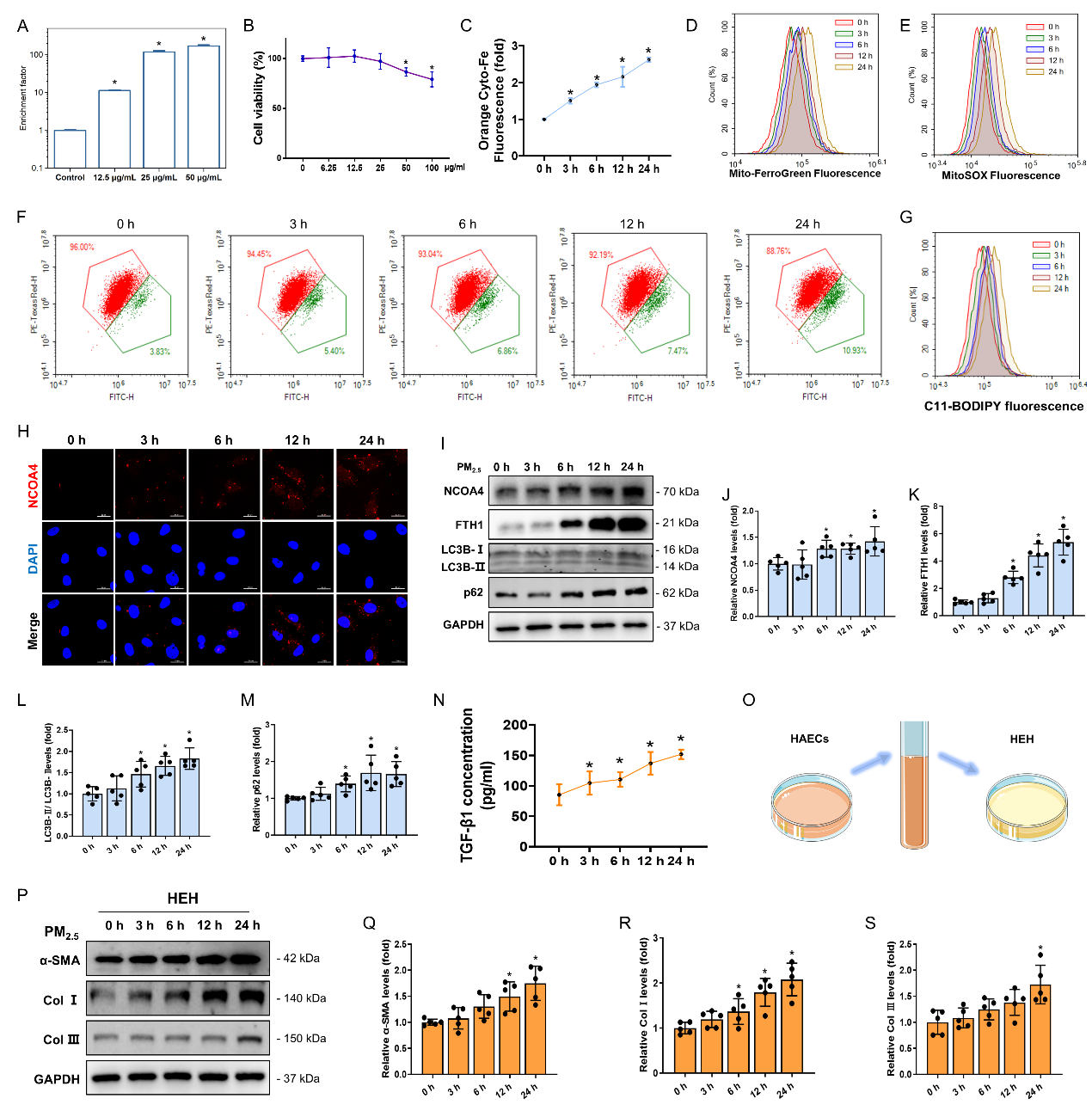


**Figure S5. PM_2.5_ induced ferritinophagy and EndMT in endothelial cells.** (A) Enrichment factors of Fe-containing particles in HAECs compared to controls, based on the particle number. (B) Cell viability. (C) Analysis of intracellular Fe^2+^ by flow cytometry. (D) Flow cytometry histogram of mitochondrial Fe^2+^. (E) Flow cytometry histogram of mtROS. (F) Flow cytometry scatter plot of mitochondrial membrane potential. (G) Flow cytometry histogram of lipidROS. (H) Representative immunofluorescence images of NCOA4 (scale bar: 20 μm). (I-M) Representative Western blot pictures and semi-quantitative analysis of NCOA4, FTH1 and LC3B-Ⅱ/LC3B-Ⅰ and p62. (N) TGF-β1 content in conditioned medium of HAECs. (O) Schematic diagram of co-culture with conditioned medium. (P-S) Representative Western blot pictures and semi-quantitative analysis of α-SMA, Collagen Ⅰ and Collagen Ⅲ. n=5. All data were expressed as mean ± standard deviation. **p* < 0.05.


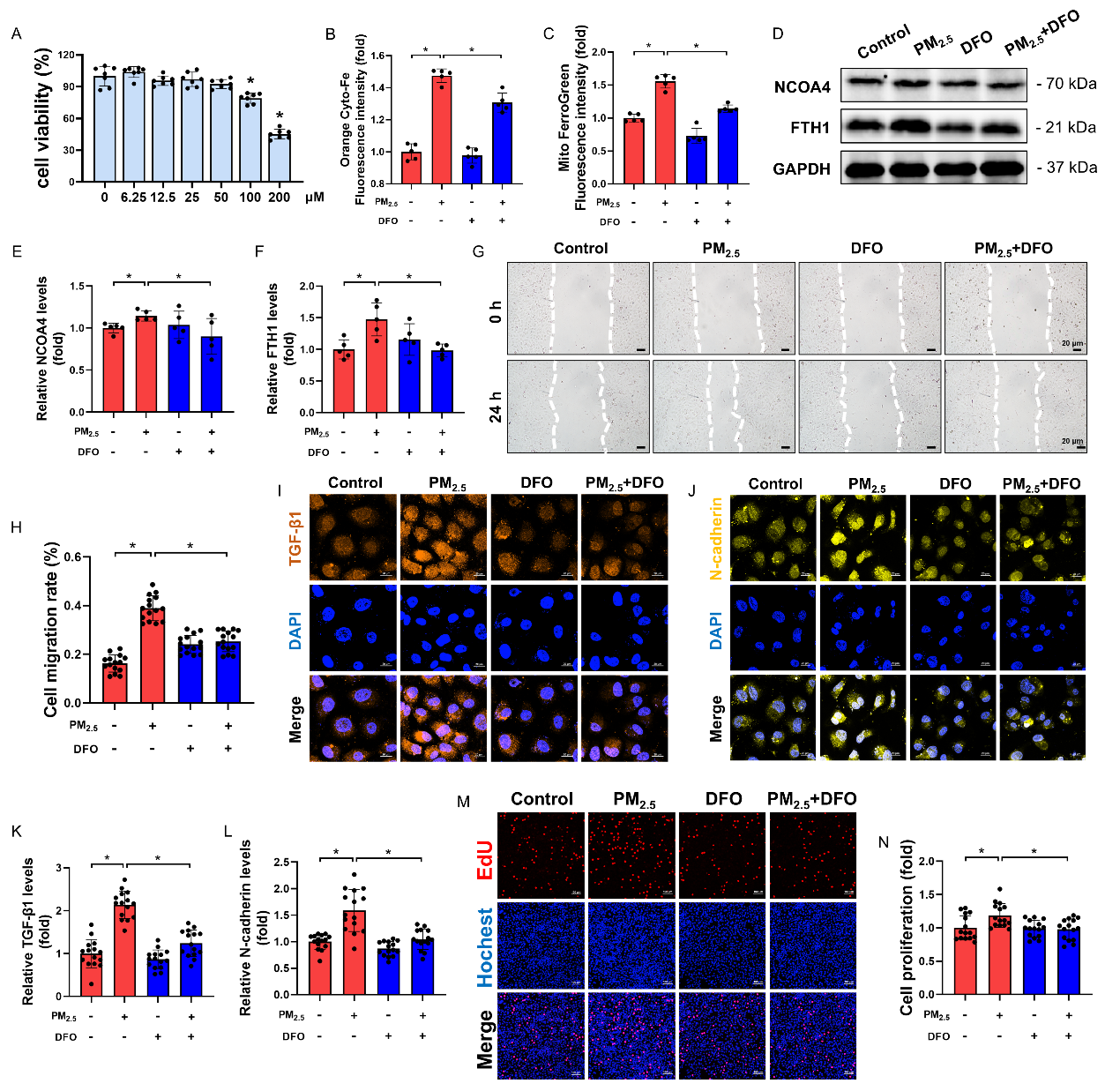


**Figure S6. Iron chelation mitigates PM_2.5_-triggered ferritinophagy and EndMT** (A) Cell viability. (B) Analysis of intracellular Fe^2+^ by flow cytometry. (C) Analysis of mitochondrial Fe^2+^ by flow cytometry. (D-F) Representative Western blot pictures and semi-quantitative analysis of NCOA4 and FTH1. (G) Representative images of cell migration assay (scale bar: 20 μm). (H) Semi-quantitative analysis of cell migration rate. (I-J) Representative immunofluorescence images of TGF-β1 and N-cadherin proteins (scale bar: 20 μm). (K-L) Semi-quantitative analysis of TGF-β1 and N-cadherin. (M) Representative fluorescence images of EdU staining (scale bar: 20 μm). (N) Semi-quantitative analysis of the degree of HEH proliferation. n=5. All data were expressed as mean ± standard deviation. **p* < 0.05.


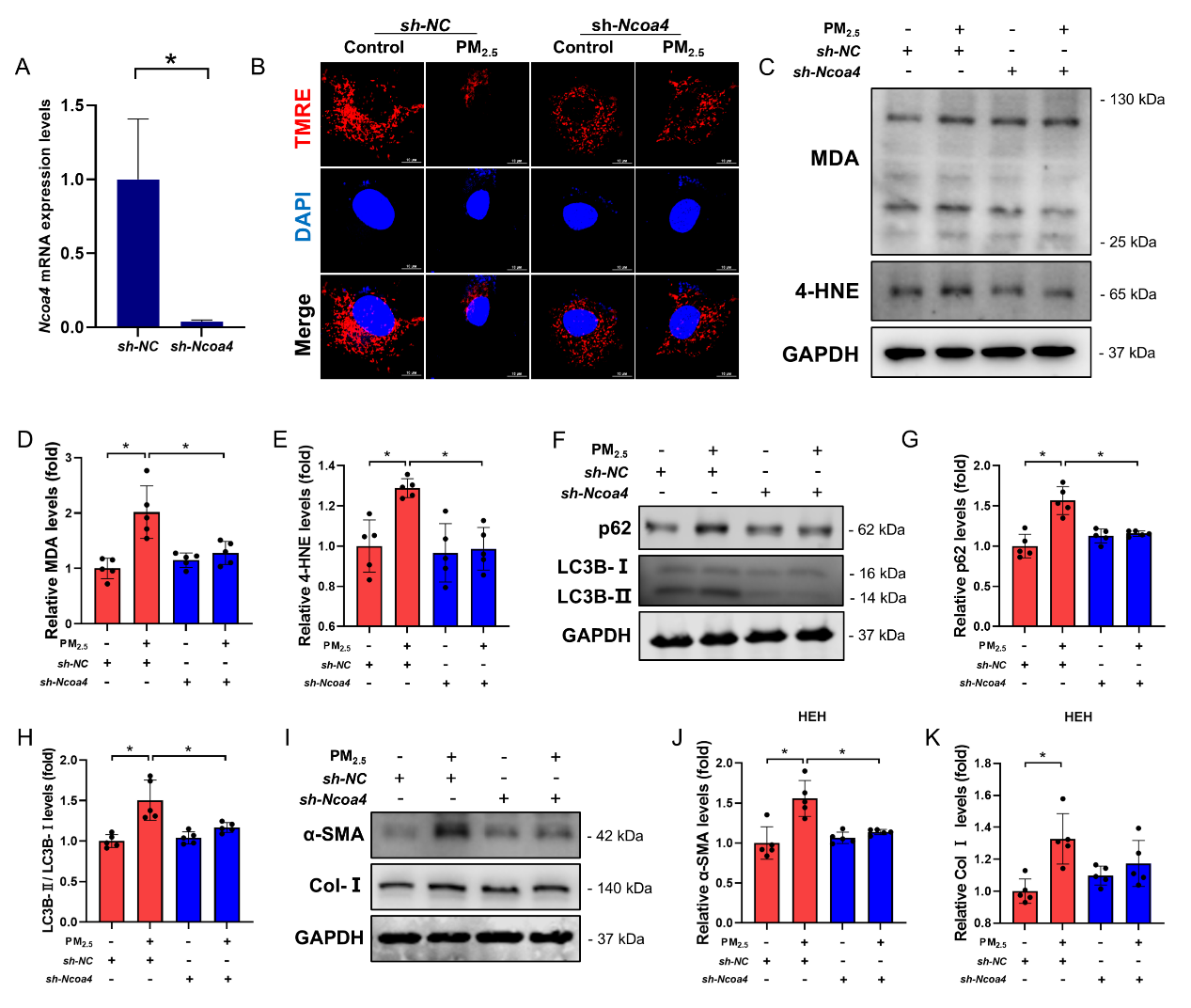


**Figure S7. PM_2.5_ regulated ferritinophagy via NCOA4.** (A) The mRNA expression of *Ncoa4*. (B) Representative fluorescence images of mitochondrial membrane potential (scale bar: 10 μm). (C-E) Representative Western blot pictures and semi-quantitative analysis of MDA and 4-HNE. (F-H) Representative Western blot pictures and semi-quantitative analysis of p62 and LC3B-Ⅱ/LC3B-Ⅰ. (I-K) Representative Western blot pictures and semi-quantitative analysis of α-SMA and Collagen Ⅰ. n=5. All data were expressed as mean ± standard deviation. **p* < 0.05.


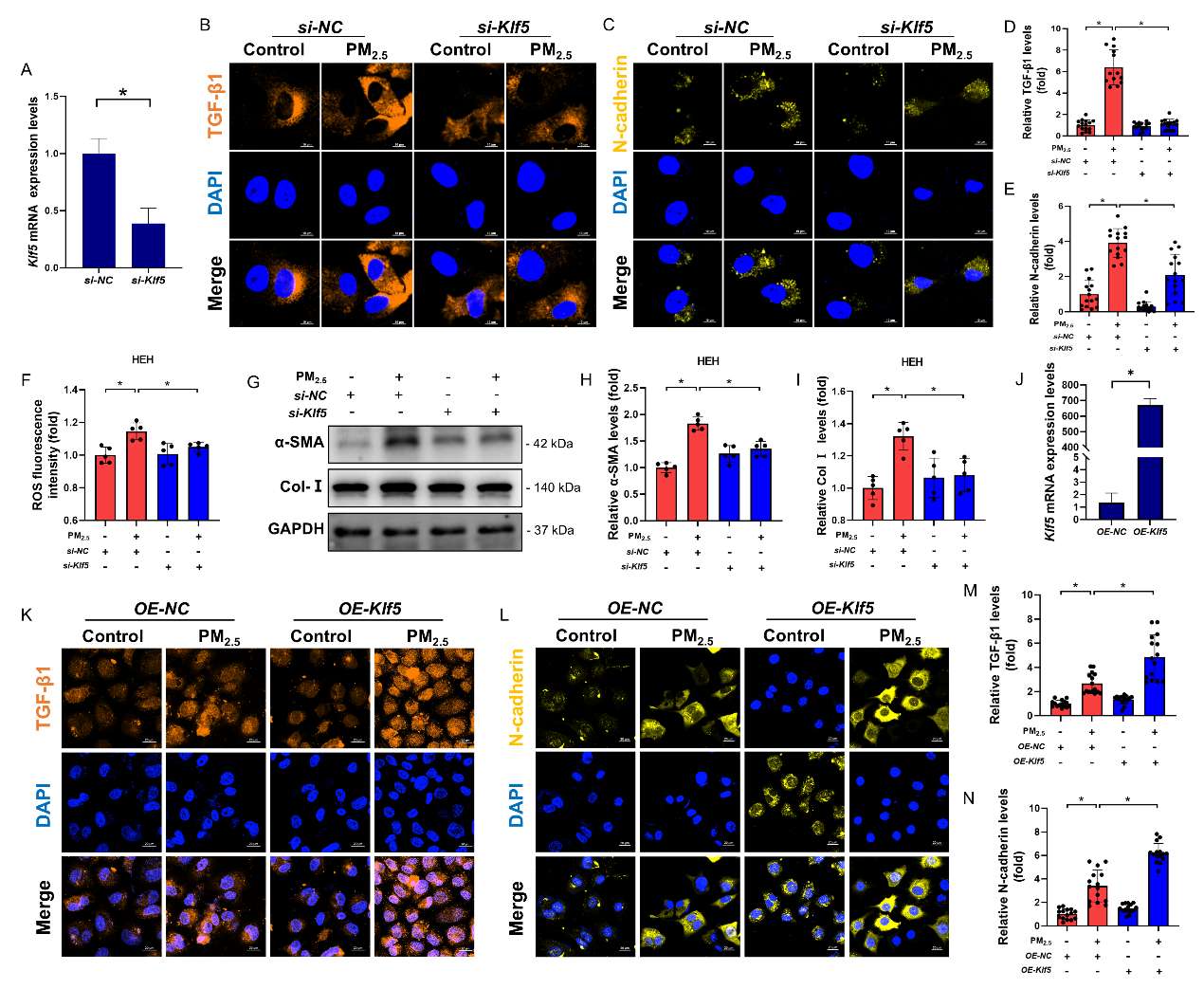


**Figure S8. PM_2.5_ evoked transcription factor KLF5 to promote *Tgf-β1* transcription.** (A) The mRNA expression of *Klf5*. (B-C) Representative immunofluorescence images of TGF-β1 and N-cadherin proteins (scale bar: 10 μm). (D-E) Semi-quantitative analysis of TGF-β1 and N-cadherin. (F) Analysis of ROS content by flow cytometry. (G-I) Representative Western blot pictures and semi-quantitative analysis of α-SMA and Collagen Ⅰ. (J) The mRNA expression of *Klf5*. (K-L) Representative immunofluorescence images of TGF-β1 and N-cadherin proteins (scale bar: 20 μm). (M-N) Semi-quantitative analysis of TGF-β1 and N-cadherin. n=5. All data were expressed as mean ± standard deviation. **p* < 0.05.


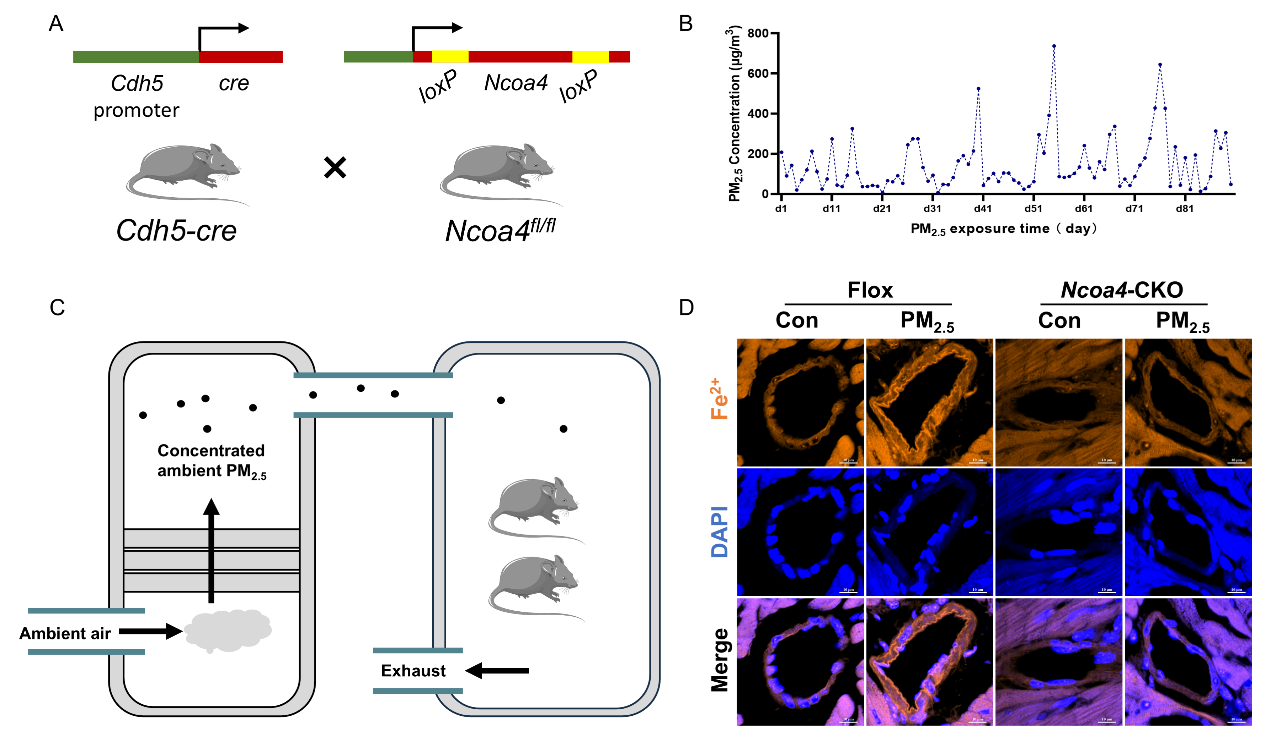


**Figure S9. PM_2.5_ exposure in mice.** (A) Generation of *Ncoa4* endothelial cell conditional knockout mice. (B) PM_2.5_ average daily exposure concentration. (C) Whole-body PM_2.5_ exposure device. (D) PM_2.5_ induced iron overload in mice cardiac endothelial cells. n=6.

**Supplementary Video**

**Supplementary Video 1.** Conformational changes of Fe_3_O_4_ nanoparticles and NCOA4 protein during their interaction process.

**Supplementary tables**

**Table S1. Operating conditions for spICP-TOF-MS**

| Operation | Conditions |
| --- | --- |
| Plasma power | 1550 W |
| Nebulizer liquid flow rate | 0.02 mL/min |
| Nebulizer gas flow rate | 0.64 L/min |
| Additional gas flow | 77.32 % |
| Attenuated masses | 32, 35, 40, 40.3 Th |
| Integration time | 2 ms |
| Transport efficiency | 40.6% |
| Mass res power 238U | 3000 |
| Auxiliary gas flow rate | 0.79 L/min |
| Cooling gas flow rate | 14.01 L/min |
| TOF detector type | Microchannel plate detector |

**Table S2. Particle mass detection limits for 22 elements detected by spICP-TOF-MS**

| **Element** | **Isotope** | **Mass detection limit (g)** |
| --- | --- | --- |
| Na | 23Na | 3.9×10^-16^ |
| Mg | 24Mg | 2.8×10^-15^ |
| Al | 27Al | 3.0×10^-16^ |
| K | 39K | 2.4×10^-14^ |
| Ca | 44Ca | 2.8×10^-15^ |
| Ti | 48Ti | 5.8×10^-17^ |
| V | 51V | 6.4×10^-17^ |
| Cr | 52Cr | 5.9×10^-17^ |
| Fe | 54Fe | 6.9×10^-16^ |
| Mn | 55Mn | 2.9×10^-17^ |
| Co | 59Co | 5.3×10^-17^ |
| Ni | 60Ni | 1.5×10^-16^ |
| Cu | 63Cu | 5.7×10^-17^ |
| Zn | 66Zn | 1.1×10^-16^ |
| Sr | 88Sr | 1.4×10^-17^ |
| Zr | 90Zr | 2.9×10^-17^ |
| Nb | 93Nb | 1.8×10^-17^ |
| Sn | 120Sn | 2.8×10^-17^ |
| Sb | 121Sb | 3.0×10^-17^ |
| Ba | 138Ba | 9.5×10^-18^ |
| W | 184W | 2.4×10^-17^ |
| Pb | 208Pb | 1.0×10^-17^ |

**Table S3. Antibodies used in the study**

| **Antibodies** | **Manufacturer** | **Catalog order numbers** | **Dilutions** |
| --- | --- | --- | --- |
| Mouse anti-NCOA4 monoclonal antibody | Santa Cruz | sc-373739 | WB: 1:200 |
| Rabbit anti-NCOA4 polyclonal antibody | Invitrogen | PA5-115626 | IF: 1:100 |
| Rabbit anti-FTH1 monoclonal antibody | Cell Signaling Technology | 4393 | WB: 1:1000  IF: 1:100 |
| Rabbit anti-p62 monoclonal antibody | Abcam | ab109012 | WB: 1:1000 |
| Rabbit anti-LC3A/B monoclonal antibody | Cell Signaling Technology | 12741s | WB: 1:1000  IF: 1:100 |
| Rabbit anti-TGF-β1 monoclonal antibody | Abcam | ab215715 | WB: 1:1000  IF: 1:100 |
| Rabbit anti-Vimentin monoclonal antibody | Cell Signaling Technology | 5741 | WB: 1:1000 |
| Rabbit anti-N-Cadherin polyclonal antibody | Abcam | ab18203 | IF: 1:100 |
| Rabbit anti-VE-cadherin Polyclonal antibody | Invitrogen | WE324566 | WB: 1:1000  IF: 1:100 |
| Rabbit anti-KLF5 monoclonal antibody | Cell Signaling Technology | 40674 | WB: 1:1000  IF: 1:100 |
| Mouse anti-α-SMA monoclonal antibody | Abcam | ab7817 | WB: 1:1000  IF: 1:100 |
| Rabbit anti-Collagen I polyclonal antibody | Abcam | ab254113 | WB: 1:1000  IHC: 1:100 |
| Rabbit anti-Collagen Ⅲ monoclonal antibody | Abcam | ab184993 | WB: 1:1000 |
| Rabbit anti-GAPDH monoclonal antibody | Cell Signaling Technology | 5174 | WB: 1:1000 |
| Rabbit anti-α-Tubulin monoclonal antibody | Cell Signaling Technology | 5335 | WB: 1:1000 |
| Rabbit anti-Lamin B1 monoclonal antibody | Cell Signaling Technology | 13435 | WB: 1:1000 |

**Table S4. Primers used for CHIP Assays in this study**

|  | **Sense (5'-3')** | **Antisense (5'-3')** |
| --- | --- | --- |
| Site #1 | ACATGGCCTACTCCCTTCCT | CCCCCGTGAAGCTGATATCC |
| Site #2 | AGATAAGACGGTGGGAGCCT | TGGGACCACACCTGGAAATG |
| Site #3 | CTGGAGATCGGCCTGCTG | ACCCCGAGGTCCTAGAAAGG |

**Methods**

***Docking and Molecular Dynamics Simulations***

The structures of Fe_3_O_4_ nanoparticles were obtained from the Materials Project database (<https://legacy.materialsproject.org/>) and optimized via molecular dynamics simulations to reflect their behavior in an aqueous environment. The initial conformations were refined through energy minimization and equilibration steps using GROMACS. The NCOA4 protein structure was derived from the AlphaFold3 ^1,2^ predicted model (<https://www.uniprot.org/uniprotkb/Q13772/entry>). The Fe_3_O_4_ nanoparticles and NCOA4 protein were placed in a simulation box containing explicit water molecules. Counterions were introduced to neutralize the system, and the CHARMM36 ^3^ force field was applied to describe the protein and water molecules, while the Interface force field, compatible with CHARMM36, was used for the Fe_3_O_4_ nanoparticles.

Molecular dynamics simulations followed a standard protocol. Initially, energy minimization was performed to eliminate steric clashes and potential energy barriers. This was followed by equilibration in the NVT and NPT ensembles to stabilize the system's temperature and pressure. A 100 ns production run was conducted under periodic boundary conditions to obtain trajectory data for further analysis. Binding free energies were calculated using the molecular mechanics-Poisson-Boltzmann surface area (MM-PBSA) method via g_mmpbsa ^4^. The interaction energy analysis included quantification of van der Waals and electrostatic contributions. Structural stability and conformational changes were assessed by analyzing root mean square deviation (RMSD), radius of gyration (Rg), and solvent-accessible surface area (SASA).

***Doppler ultrasound assessment***

Mice were anesthetized, and echocardiographic images were obtained using a Vevo 2100 Ultra-High-Resolution Small Animal Ultrasound Imaging System (FUJIFILM Visualsonic, Canada), M-mode was used to detect left ventricle wall thickness, internal dimensions, and left ventricle volume during systole and diastole. Fraction shorting (FS) and ejection fraction (EF) were calculated to reflect contractile function. All parameters were determined in at least 3-5 cardiac cycles.

***Cell culture and treatment***

Human aortic endothelial cells (HAECs) were purchased from the American Type Culture Collection (ATCC). The human embryonic heart fibroblast cell line (HEH) was obtained from the Kunming Cell Bank of Chinese Academy of Sciences. HAECs and HEH were cultured in a 100-mm culture dish in DMEM/F12 (VivaCell, China) containing 10% fetal bovine serum (Gibco, USA) and 1% penicillin/streptomycin solution in 37℃, 5% CO_2_ incubator. The cells were inoculated and converged to 70-80% density before treatment. HAECs were exposed to different concentrations of PM_2.5_-containing medium or for various exposure times. The HAECs were pretreated with deferoxamine (DFO) for 2 h before exposure to PM_2.5_. HEH were cultured by conditioned medium of HAECs after treating with PM_2.5_.

***Assessment of cell viability***

Cell Counting Kit-8 was employed to determine the cytotoxicity of PM_2.5_ (CCK8; Dojindo Molecular Technologies, CK04). Briefly, HAECs were inoculated into 96 well plates and cultured in a 37℃ incubator for 24 h. Afterward, the cells were treated with DMEM/F12 (control) or DMEM/F12 containing different concentrations (6.25, 12.5, 25, 50, 100 μg/mL) of PM_2.5_ for 24 h, respectively. Finally, the cells were incubated in DMEM/F12 containing 10% CCK8 reagent for 1 h. The cell viability was calculated by recording the optical density (OD) at 450 nm by a microplate reader (Synergy HT; Bio Tek, Winooski, VT, USA). Similarly, the HAECs were treated with DFO at a series of concentrations (0, 6.25, 12.5, 25, 50, 100, 200 μM) for 24 h. Then cell viability was measured by CCK-8 kit.

***Cell transfection***

For transfection experiments, lentivirus of *Ncoa4* was added to HAECs and 10 μg/mL of Polybrene was added to promote the efficiency of virus-infected cells. After 12 h, the virus-containing medium was replaced with fresh complete medium. Puromycin was used to screen virus-infected HAECs to establish stable *Ncoa4* knockdown HAECs cell lines. *Klf5* siRNA (20 μM) was pre-transfected into the HAECs using Lipofectamine™ 3000 (Invitrogen, USA) for 24h in vitro experiments. To generate KLF5-overexpressing cells, HAECs were likewise transduced with a *Klf5*-overexpressing lentivirus in the presence of 10 µg/mL Polybrene. After 12 h the medium was refreshed, and cells were selected with 1 µg/mL puromycin for 2 days to establish stable KLF5-overexpressing HAEC lines.

***Immunofluorescence and Immunohistochemical staining***

Paraffin-embedded sections of heart tissues or HAECs were analyzed by immunofluorescence or histochemistry for the expression of TGF-β1, N-cadherin, NCOA4, FTH1, KLF5, α-SMA, and Col Ⅰ. Immunofluorescence methods: paraffin sections were dewaxed, hydrated, and antigenically repaired. After treatment, HAECs were inoculated into confocal petri dishes and fixed with 4% paraformaldehyde (Solarbio, China) for 30 min at room temperature. Sections or cells were permeabilized with 0.5% Triton X-100 for 30 min and blocked in 10% goat serum (ZSGB-BIO, ZLI-9021) at 37℃ for 2 h. Appropriately diluted primary antibody was added dropwise and incubated at 4℃ overnight. Fluorescent secondary antibody was added dropwise and incubated at 37℃ for 1 h. DAPI was used to stain the nuclei and images were captured using a scanning confocal microscope (Nikon Eclipse Ti2, Japan). Multiplex immunohistochemical method: Multiplex fluorescence immunohistochemical staining kit (Absin, China) was used for staining. Briefly, paraffin sections were incubated with primary antibody, incubated with HRP secondary antibody dropwise, and then fluorescence staining was performed to amplify the signal. Re-antigen repair, sealing, and repeating the above steps until all antibodies were bound, sealing the section for observation. Immunohistochemistry: The kit (BOSTER, USA) was used for the analysis. The steps before secondary antibody incubation were the same as immunofluorescence, and the sections were incubated with biotinylated goat anti-rabbit IgG dropwise at 37℃ for 20 minutes. The nuclei were stained with nuclear solid red, and the slices were sealed for observation. All antibody information was presented in Table S3.

***Determination of Fe^2+^ levels***

Intracellular and mitochondrial iron content of HAECs was determined using FerroOrange (1 μM) and Mito-FerroGreen (5 μM) probes (DojinDo, Japan). The treated cells were incubated with the probe at 37℃ for 30 min under dark conditions. Cells were then collected for fluorescence intensity assessment by flow cytometry (Becton Dickinson, USA). FerroOrange (1 μM) probe was used to incubate the deparaffinized sections of heart tissues under dark conditions for 30 min, and after sealing the sections with drops of DAPI, the Fe^2+^ content was observed by confocal microscopy.

***Lipid ROS and mitochondrial ROS assay***

ROS in lipids were detected with the molecular probe BODIPY™581/591 C11 (Invitrogen, USA). MitoSOX (Invitrogen, USA) fluorescent dye was used to detect mitochondrial ROS production. Cells were treated and incubated with 5 μM BODIPY™581/591 C11 or 5 μM MitoSOX probe for 30 min under dark conditions. Fluorescence signals were acquired by flow cytometry and Image J was used to measure relative fluorescence intensity.

***Analysis of mitochondrial membrane potential (MMP)***

Cellular MMP levels were detected by the JC-1 probe (Beyotime, China) and the TMRE probe (Beyotime, China), which selectively penetrates mitochondria and reversibly switches from red to green as MMP decreases. The TMRE probe could aggregate in intact mitochondria, and decreased MMP was accompanied by decreased TMRE accumulation. The treated cells were incubated with 10 mg/L JC-1 or 10 μM TMRE for 20 min, and the cells were collected and analyzed for fluorescence intensity by flow cytometry or observed under a laser confocal microscope.

***Measurement of TGF-β1 levels***

The content of TGF-β1 in conditioned medium of HAECs was assayed using a TGF-β1 ELISA Kit (Multi Sciences, China) following the manufacturer's instructions. The specific content of TGF-β1 was expressed as pg/mL.

***Cell migration assay***

Inoculate the HAECs in a 6 well plate. When the cells are full grown, draw a plumb line perpendicular to the well plate. Discard the old medium and rinse off the scratched down cells by washing gently with PBS three times. Images were taken with a microscope as a 0 h control. After the cells were exposed to PM_2.5_ for 24 h, the width of the scratch at the same location was observed and photographed.

***Transmission electron microscopy (TEM) assays***

The heart tissues (1 mm^3^) or HAECs after treatment were placed in pre-cooled 2.5% glutaraldehyde solution and fixed at 4℃ for 3 h. Then the samples were washed with 0.1 M phosphate buffer (PB, Coolaber, SL1326), embedded in 2% agarose gels, fixed in 4% osmium tetroxide solution (Sigma-Aldrich, 251755) for 1 h, dehydrated with graded amounts of ethanol (50%, 70%, 80%, and 90%) and acetone, and embedded in epoxy resin (Sigma-Aldrich, 45347). Ultrathin sections (50-60 nm) were cut with an ultrathin microtome, stained with aqueous uranyl acetate (5%) and aqueous lead citrate (2%), air-dried, and visualized by a TEM (JEOL JEM2100, Tokyo, Japan).

***Extraction of nuclear and cytoplasmic proteins***

The protein extraction kit (Beyotime, China) was used to extract nuclear or cytoplasmic proteins from HAECs. Briefly, HAECs in the culture dish were washed with PBS and scraped off with a cell scraper. After centrifugation, cytoplasmic protein extraction reagent A with PMSF was added to the cell sediment, which was completely dispersed by vigorous shaking. The supernatant containing cytoplasmic protein was obtained by shaking and centrifugation after the addition of cytoplasmic protein extraction reagent B. PMSF-conjugated nucleoprotein extraction reagent was added to the remaining precipitate, and the supernatant containing nucleoprotein was obtained by shaking and centrifugation.

***Dual-luciferase reporter assay***

Wild-type and mutant reporter gene plasmids were constructed with the vector PGL4.10, and a transcription factor overexpression plasmid was constructed with the vector pCDNA3.1. One day before transfection, HEK293T cells were inoculated in 96 well plates at 2×10^4^ cells/well. The cells were co-transfected with the reporter gene plasmid or the transcription factor expression plasmid. After 48 h of transfection, the old medium was aspirated, 100 μL of passive lysis buffer was added to each well, and the cells were lysed for 15 min at room temperature on a shaker. The relative light unit was determined by mixing the cell lysis buffer (20 μL) and luciferase assay reagent (100 μL). The background, firefly fluorophore, and renilla fluorophore levels were read separately, and the ratio was calculated by subtracting the background value from each of the two fluorophore values: (firefly fluorophore-background) / (renilla fluorophore-background).

***Chromatin immunoprecipitation (ChIP)-qPCR***

Pierce^TM^ magnetic ChIP Kit (ThermoFisher, USA, Cat: 26157) were used in this study according to the manufacturer’s instructions. In brief, cells were crosslinked by 1% formaldehyde and chromatin was extracted and sheared by Ultrasonic crusher. Samples were immunoprecipitated with anti-KLF5 antibody 4℃ overnight. The immunoprecipitated DNA was purified and analyzed by qPCR with primers. The primer sequences were listed in Supplementary Table S4.

***EdU cell proliferations***

EdU is a thymine nucleoside analog that could substitute for thymine during the period of DNA replication and infiltrate into the DNA molecule being synthesized. Cell-Light EdU Apollo567 In Vitro Kit was used to assess the proliferation of HEH. After adding the complete medium containing EdU for 2 h, the cells were fixed for 30 min and stained using a fluorescent dye that bound to EdU to visualize the EdU markers. Images of the stained cells were acquired using the confocal microscope.

***Histopathological analysis***

Paraffin sections of mice heart tissues were stained with hematoxylin-eosin staining (H&E) and Masson trichrome staining to observe the changes in tissue morphology and fibrosis. Wheat germ agglutinin (WGA) (Sigma, USA) was used for fluorescent staining of cardiac sections to determine cardiomyocyte cross-sectional area. The slices were imaged by an automatic slice scanning system (3Dhistech, Hungary).

***Western blotting***

The proteins extracted from the cells were quantified by BCA protein assay kit (KeyGEN Biotech, China). The proteins were loaded in equal amounts, separated by SDS-PAGE gels, and transferred to NC membranes. After blocking in TBS with skim milk (5%), the membranes were incubated at 4 ℃ overnight with primary antibodies, including NCOA4, FTH1, TGF-β1, Vimentin, VE-cadherin, KLF5, α-SMA, Col Ⅰ, Col Ⅲ, MDA, 4-HNE, p62, LC3B, LaminB1, and GAPDH. Thereafter, a secondary antibody was used to incubate the membranes. The ChemiDoc MP Imaging System (Bio-Rad, USA) or the LI-COR Odyssey ® CLx Infrared Imaging System were employed to detect the signals of protein bands. Image J was used to analyze the density of the detected protein bands.

***Statistical analysis***

GraphPad Prism (v.9.0, GraphPad Software, La Jolla, CA, USA) was used for statistical analysis and plotting. All data were expressed as mean ± standard deviation. Multi-group comparisons were performed using the one-way analysis of variances with Bonferroni correction. *p* <0.05 was considered a statistically significant difference.

**References**

1. Roy, R. & Al-Hashimi, H. M. AlphaFold3 takes a step toward decoding molecular behavior and biological computation. *Nat. Struct. Mol. Biol.* **31**, 997–1000 (2024).

2. Wee, J. & Wei, G.-W. Benchmarking AlphaFold3’s protein-protein complex accuracy and machine learning prediction reliability for binding free energy changes upon mutation. *ArXiv* arXiv:2406.03979v1 (2024).

3. Huang, J. & MacKerell, A. D. CHARMM36 all-atom additive protein force field: validation based on comparison to NMR data. *J. Comput. Chem.* **34**, 2135–2145 (2013).

4. Kumari, R., Kumar, R., Open Source Drug Discovery Consortium & Lynn, A. g_mmpbsa--a GROMACS tool for high-throughput MM-PBSA calculations. *J. Chem. Inf. Model.* **54**, 1951–1962 (2014).
